# Stimulation of Vibratory Urticaria-Associated Adhesion-GPCR, EMR2/ADGRE2, Triggers the NLRP3 Inflammasome Activation Signal in Human Monocytes

**DOI:** 10.3389/fimmu.2020.602016

**Published:** 2021-01-08

**Authors:** Kuan-Yu I, Wen-Yi Tseng, Wen-Chih Wang, Siamon Gordon, Kwai-Fong Ng, Hsi-Hsien Lin

**Affiliations:** ^1^ Department of Microbiology and Immunology, College of Medicine, Chang Gung University, Taoyuan, Taiwan; ^2^ Division of Rheumatology, Allergy and Immunology, Chang Gung Memorial Hospital-Keelung, Keelung, Taiwan; ^3^ Sir William Dunn School of Pathology, University of Oxford, Oxford, United Kingdom; ^4^ Department of Anatomic Pathology, Chang Gung Memorial Hospital-Linkou, Taoyuan, Taiwan

**Keywords:** adhesion G protein-coupled receptor, inflammasome, NLRP3, signaling, pathogen-associated molecular patterns

## Abstract

EMR2/ADGRE2 is an adhesion G protein-coupled receptor differentially expressed by human myeloid cells. It modulates diverse cellular functions of innate immune cells and a missense EMR2 variant is directly responsible for vibratory urticaria. Recently, EMR2 was found to activate NLRP3 inflammasome in monocytes *via* interaction with FHR1, a regulatory protein of complement Factor H. However, the functional involvement of EMR2 activation and its signaling mechanisms in eliciting NLRP3 inflammasome activation remain elusive. In this study, we show that EMR2-mediated signaling plays a critical role in triggering the activation (2^nd^) signal for the NLRP3 inflammasome in both THP-1 monocytic cell line and primary monocytes. Stimulation of EMR2 by its agonistic 2A1 monoclonal antibody elicits a Gα_16_-dependent PLC-β activation pathway, inducing the activity of downstream Akt, MAPK, NF-κB, and Ca^2+^ mobilization, eventually leading to K^+^ efflux. These results identify EMR2 and its associated signaling intermediates as potential intervention targets of NLRP3 inflammasome activation in inflammatory disorders.

## Introduction

Proteolytic conversion of procaspase-1 to active caspase-1 and the subsequent processing and secretion of inflammatory IL-1β and IL-18 are the hallmarks of inflammasome activation (1, 2). Although it is a critical immune response to microbial infection and tissue injury, dysregulated inflammasome activities are often linked to pathological manifestations such as autoinflammatory, autoimmune, and metabolic disorders as well as cancer (3–5). The NLRP3 inflammasome is a large assembly of multimeric NLRP3, ASC and procaspase-1, and represents the principal inflammasome activated in innate immune cells through recognition of pathogen-associated molecular patterns (PAMPs) and/or damage-associated molecular patterns (DAMPs) (6, 7). The canonical NLRP3 inflammasome activation pathway is normally mediated by a two-signal mechanism; the priming (1^st^) signal is induced by PAMPs and the activation (2^nd^) signal elicited by diverse cellular stressors or DAMPs such as extracellular ATP, pore-forming toxins and particulates (6, 8). While the priming signal is mostly transmitted by pattern recognition receptors (PRRs), the activation signal can be triggered by an increasing number of different cell surface proteins including G protein-coupled receptors (GPCRs) (8, 9). As GPCRs are extensively-proven drug targets, identification and characterization of novel GPCRs involved in NLRP3 inflammasome activation not only can advance our understanding of this important immune reaction, but also be exploited for potential therapeutics of inflammasome-associated disorders (10).

EMR2/ADGRE2 is a human-specific paralogue of the well-known mouse tissue macrophage (Mφ)-specific F4/80 antigen (Ag) (11). Both EMR2 and F4/80 belong to the ADGRE/EGF-TM7 subfamily of adhesion GPCRs (aGPCRs) that is distinguished by multiple extracellular epidermal growth factor (EGF)-like domains (12–14). Unlike F4/80, however, EMR2 is more widely expressed and differentially regulated in cells of the myeloid lineage including monocytes, Mφ, neutrophils (Nφ), and myeloid dendritic cells (DCs) (13, 15). EMR2 expression is up-regulated restrictedly within inflamed tissues and its levels on Nφ are positively correlated with the severity and overall mortality of patients of systemic inflammatory response syndrome (SIRS) and liver cirrhosis (16, 17). These findings indicated an immune regulatory function for EMR2. Indeed, EMR2 activation has been shown to potentiate adhesion, migration and anti-microbial activities of Nφ in response to diverse inflammatory stimuli (18–20). A missense EMR2-pC492Y variant was identified recently as the cause of familial vibratory urticaria (VU), a rare autosomal dominant dermal allergic disorder resulting from vibration-induced mast cell activation (21).

We have previously identified dermatan sulphate (DS) as an endogenous glycosaminoglycan ligand of EMR2 (22). DS-EMR2 interaction plays a potential role in recruiting monocytes to the inflamed synovium in rheumatoid arthritis (RA) (23). Interaction of the VU-inducing EMR2-pC492Y variant with DS or a specific 2A1 monoclonal antibody (mAb) sensitizes mast cells for hyper-degranulation upon vibratory stimulation, indicating a mechanosensing role for EMR2 (21). Our earlier results revealed that 2A1-induced EMR2 activation in monocytic cells lead to Mφ-like phenotypes and enhanced production of IL-8 and TNF *via* a Gα_16_-dependent pathway activating the downstream effectors, including phospholipase C (PLC)-β, PI3K, Akt, MAPK, and NF-κB (24). Interestingly, 2A1-induced EMR2 signaling occurred only when the mAb was immobilized on the culture plates (24). In a recent study, complement Factor H-related protein 1 (FHR1) was shown to function both as a sensor of necrotic cells and a specific serum ligand of EMR2 (25). Consequently, FHR1 was shown to mark necrotic regions of vasculopathies such as anti-neutrophil cytoplasmic antibody-associated vasculitis (AAV) and atherosclerosis, and concomitantly triggered EMR2-mediated NLRP3 inflammasome activation in monocytes *via* a Gβγ-dependent PLC signaling pathway. Peculiarly, the FHR1-induced EMR2 activation was induced only in the presence of normal human serum (NHS) and immobilized FHR1 (25). Taken together, EMR2 is a human myeloid-restricted aGPCR whose activation and signaling is involved in distinct innate immune functions including NLRP3 inflammasome activation.

In this study, we show that 2A1-induced EMR2 signaling plays a critical role in triggering the NLRP3 inflammasome activation (2^nd^) signal, due to the intracellular K^+^ efflux evoked *via* a Gα_16_-dependent PLC-β activation and Ca^2+^ mobilization. EMR2 and its downstream signaling effector molecules hence represent novel GPCR-associated targets for intervention of NLRP3 inflammasome activation in relevant inflammatory disorders.

## Materials and Methods

### Reagents and Antibodies

All chemicals and reagents were purchased from Sigma (St. Louis, MO, USA) and Invitrogen (CA, USA) unless specified otherwise. ATP, N-acetyl-L-cysteine (NAC), diphenyleneiodonium chloride (DPI), U73122, BAPTA-AM, Glyburide, Bay 11-7082, and sp600125 were all from Sigma. Monosodium urate (MSU) was from Invitrogen. Gallein was from Tocris Bioscience (Bristol UK). U0126 was from Promega (Madison, WI, USA). SB203580 was from Cayman Chemical (Ann Arbor, MI, USA). z-YVAD-fmk (ALX-260-074) and Ac-YVAD-CHO (ALX-260-027) were from Enzo Life Sciences (Farmingdale, NY, USA). Muramyl dipeptide (MDP), Pam3CSK4, FLA-ST, and LPS-B5 Ultrapure were obtained from InvivoGen (San Diego, CA, USA). AZD9056 was from MedChemExpress (Princeton, NJ, USA). Monoclonal antibodies (mAbs) used for Western blotting included: anti-caspase-1 (D3U3E) and anti-human IL-1β (D7F10) were obtained from Cell Signaling Technology (Beverly, MA, USA); anti-ASC (AL177) was from AdipoGen (San Diego, CA, USA); anti-NLRP3 (ALX-804-819) was from Enzo Life Sciences; Anti-β-actin mAb was purchased from BD Biosciences (San Jose, CA, USA). Anti-GAPDH mAb was from Proteintech (IL, USA). Abs used for cell stimulation (2A1 and mouse IgG_1_ control) and for the detection of signaling molecules have been described previously (24).

### Cell Culture and Primary Cell Isolation

THP-1 (ATCC®TIB-202™), as well as THP-1-defNLRP3 and THP-1-defASC monocytic cell lines (Invitrogen) were cultured in RPMI 1640 medium supplemented with 10% Fetal bovine serum (FBS) (Thermo HyClone), 1% L-glutamate, 1% penicillin, 1% streptomycin and 100 μg/ml Normocin (Invitrogen). For THP-1-defNLRP3 and THP-1-defASC cells, Hygromycin B Gold (10 mg/ml)(Invitrogen) was added to culture medium. All cells were cultured in a 5% CO_2_ incubator at 37°C. Ficoll-Plague PLUS (Amersham Bioscience, Ltd) gradient centrifugation was used to purify peripheral blood mononuclear cells (PBMCs) from venous blood of healthy donors as described previously (24). All procedures were approved by the Chang Gung Memorial Hospital Ethics Committee (CGMH IRB No: 201700390B0 and 202001020B0) and performed according to their guidelines. Monocytes were isolated from PBMCs by immune-magnetic separation using human CD14 MicroBeads MACS cell separation kit (Miltenyi Biotec, Inc) and cultured in complete RPMI 1640 medium. Unless otherwise specified, 12- or 6-well cell culture plates were pre-coated with appropriate mAbs (usually 10 μg/ml) in 1× PBS at 4°C for 24 h. Cells (5x10^5^~2x10^6^ cells/ml) were treated without or with lipopolysaccharide (LPS)(50-100 ng/ml) or other PAMPs as indicated for 24 h. For inhibitor treatment, cells were incubated with culture medium containing the following reagents as indicated: z-YVAD-fmk (50 μM), Ac-YVAD-CHO (50 μM), Bay117082 (10 μM), U0126 (10 μM), sp600125 (10 μM), SB203580 (10 μM), LY294002 (20 μM), TPAC-1 (10 μM), Gallein (10 μM), Glyburide (25 μg/ml), U73122 (20 μM), BAPTA-AM (10 μM), NAC (10 μM), and DPI (10 μM). Normal human serum (NHS) was derived from fresh human peripheral blood left to clot at room temperature for 30 min, followed by centrifugation at 1,500 x g for 10 min at 4°C. NHS was kept frozen in aliquots at −80°C and used at a final concentration of 5%.

### siRNA-Mediated Gene Silencing

All siRNAs used were purchased from Invitrogen. Briefly, 200 nM of gene-specific siRNAs were transfected into THP-1 cells using DharmaFECT-2 transfection reagent (GE Dharmacon) and incubated for 48 h as suggested by the manufacturer. The siRNA sequence information is listed below: NLRP3-siRNA #1: 5’-ACCGCGGUGUACGUCUUCUUCCUUU-3’, NLRP3-siRNA #2: 5’-GGAUUGAAGUGAAAGCCAAAGCUAA-3’, NLRP3-siRNA #3: 5’-UCCACCAGAAUGGACCACAUGGUUU-3’, ASC-siRNA #1: 5’-GGCUGCUGGAUGCUCUGUACGGGAA-3’, ASC-siRNA #2 ASC-siRNA: 5’-ACCCAAGCAAGAUGCGGAAGCUCUU-3’, ASC-siRNA #3: 5’-GCCUGG AACUGGACCUGCAAGGACU-3’. EMR2-specific, Gα_16_-specific, and scramble control siRNAs were used as described previously (24).

### Detection of ASC Oligomerization

THP-1 cells (4 × 10^6^ cells) were pelleted by centrifugation and resuspended in 300 μl ice-cold buffer 1 (20 mM HEPES-KOH, pH 7.5, 150 mM KCl, 320 mM sucrose, 0.01 μg/ml Aprotinin, 10 mM AEBSF, 20 mM Levamisole, 0.1 mM sodium orthovanadate (Na_3_VO_4_) and protease inhibitor mixture) and lysed by vortex vigorously for 10 s. Cell lysates were centrifuged at 520× g for 8 min at 4°C and pellets removed. Supernatants were diluted with 300 μl Buffer 2 (20 mM HEPES-KOH, 5 mM MgCl_2,_ 0.5 mM EDTA, 0.1% CHAPS) and centrifuged at 4000× g for 8 min to pellet the ASC pyroptosome (26). Next, the pellets were resuspended in 300 ml Buffer 3 (20 mM HEPES-KOH, 2 mM DSS, 5 mM MgCl_2,_ 0.5 mM EDTA), incubated at room temperature for 30 min, and pelleted again by centrifugation at 4000× g for 10 min. The cross-linked pellets were resuspended in SDS sample buffer (30 μl), separated in 12% SDS-PAGE by gel electrophoresis, and blotted using anti-ASC mAb.

### Cytokine ELISA Assay

Unless specified otherwise, cells (2 × 10^6^ cells/well) were seeded into 6-well plates pre-coated with or without 2A1 mAb in the absence or presence of various PAMPs, then incubated at 37°C for 24 h. Conditioned medium was collected by centrifugation at 1000x g for 5 min at 4°C and transferred into new 1.5 ml eppendorf tubes. The levels of human IL-8, IL-1β, and IL-18 were measured by DuoSet^®^ ELISA Development Systems (R&D System) according to the protocols suggested by the manufacturer.

### Western Blot Analysis

Cell lysates for Western blot analysis were collected at specific time points as indicated. In brief, cells were harvested by centrifugation at 1500 rpm for 5 min at 4°C, washed once with ice-cold 1× HBSS, and lysed in 100 μl ice-cold modified cell lysis buffer as described previously (24). Proteins were quantified using Bicinchoninic acid (BCA) protein assay kit (PIERCE, Rockford, USA). Proteins were denatured and separated by electrophoresis in SDS-polyacrylamide gels, and transferred to polyvinylidene fluoride (PVDF) membranes (Millipore, MA, USA). The blotted PVDF membranes were incubated in blocking buffer (5% of BSA in washing buffer) for 1 h with agitation, followed by incubation with the indicated 1^st^ Ab (2–4 μg/ml in blocking buffer) for 1 h. The membranes were washed extensively and then incubated with appropriate horseradish peroxidase (HRP)-conjugated 2^nd^ Ab (1:2000-1:5000 in blocking buffer). Following final extensive washes, bound 2^nd^ Ab was detected by chemiluminescence (ECL, Amersham Life Science Ltd or SuperSignal West Pico Plus, Pierce).

### Statistical Analysis

Quantitative analysis was based on results of at least three independent experiments unless indicated otherwise. Data were analyzed using one-way ANOVA analysis by Prism 5 software (GraphPad Software Inc., La Jolla, California, USA) and shown as means ± standard error of the mean (SEM). The statistical significance of *p* value was set at ^*^ *p*<0.05, ^**^ *p*<0.01, ^***^ *p*<0.001, ^****^ *p*<0.0001.

## Results

### Ligation and Activation of EMR2 Receptor in Human Monocytic Cells Enhance IL-1β and IL-18 Production in the Presence of PAMPs

**Figure 1:**
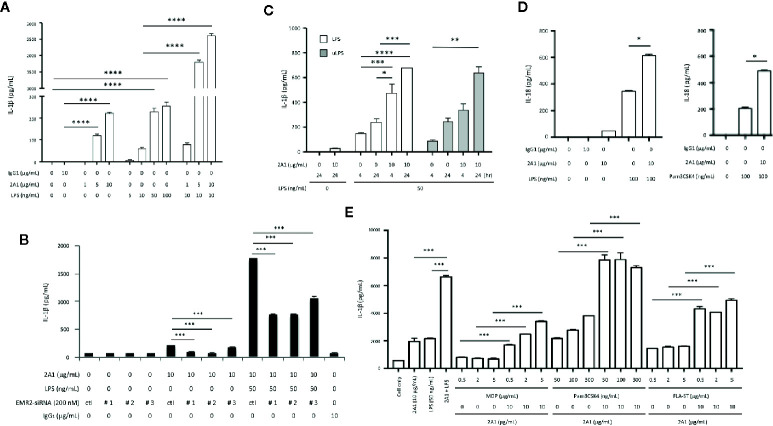
EMR2 receptor ligation in THP-1 cells by 2A1 mAb induces IL-1β and IL-18 production in the presence of PAMPs. (A–C) THP-1 cells (1x10^6^ or 5x10^5^ cells/ml) were cultured in 6-well **(A, B)** or 12-well **(C)** plates coated without or with 2A1 mAb in the absence or presence of conventional or ultrapure LPS (uLPS) for durations as indicated. When indicated, cells were transfected with EMR2-specific siRNAs (EMR2-siRNA#1, #2 or #3) to test the specific effect of 2A1-induced EMR2 activation **(B)**. Culture supernatant was collected for the detection of mature IL-1β by ELISA. Mouse IgG_1_ was included as a negative control. Ctl, scrambled siRNA control. **(D)** THP-1 cells (5x10^5^ cells/ml) in 12-well plates were treated without or with immobilized 2A1 mAb in the absence or presence of LPS or Pam3CSK4 for 24 h. Culture supernatant was collected and analyzed for IL-18 by ELISA. **(E)** THP-1 cells (2x10^6^ cells/ml) in 6-well plates were treated without or with immobilized 2A1 mAb in the presence of different TLR and NLR ligands (MDP, Pam3CSK4, and FLA-ST) as indicated for 24 h, then the level of IL-1β in culture supernatant was determined by ELISA. Data are means ± SEM of five independent experiments performed in triplicate and analyzed by one-way ANOVA. ^*^ *p*<0.05, ^**^ *p*<0.01, ^***^ *p*<0.001, ****p<0.0001.

To investigate the role of EMR2-induced signaling in inflammasome activation, THP-1 monocytic cells were cultured on plates coated with EMR2-specific 2A1 mAb in the absence or presence of various PAMPs. Low but significant levels of IL-1β were noted when cells were stimulated with 2A1 or LPS individually. However, much higher IL-1β levels were detected when cells were treated simultaneously with 2A1 and LPS, indicating a synergistic effect (**Figure 1A**). By contrast, neither the isotype control nor the soluble 2A1 mAb had any effect on IL-1β production (**Supplementary Figure 1A**). Importantly, the specificity of 2A1-induced EMR2 activation in up-regulating IL-1β was verified in cells transfected with EMR2-specific siRNAs (**Figure 1B** and **Supplementary Figure 1B**). These results were in line with our previous data and hence all following experiments were performed using immobilized 2A1 mAb (24). Similar 2A1-induced IL-1β up-regulation was obtained in cells treated with conventional or ultrapure LPS, and this effect was both concentration- and time-dependent; ranging from 5–10 μg/ml of 2A1 mAb and starting as early as 4 h of incubation (**Figures 1A, C** **and** **Supplementary Figure 1C**). In parallel, significantly higher IL-18 levels were produced by cells co-stimulated with 2A1 plus LPS or 2A1 plus Pam3CSK4 (TLR2/TLR1 ligand) than those treated with the stimulus alone (**Figure 1D**). Finally, enhanced levels of IL-1β were also identified in cells co-stimulated with 2A1 and various TLR and NLR ligands including Pam3CSK4, FLA-ST (TLR5 ligand) and MDP (NOD2 ligand)(**Figure 1E**). These results suggest that EMR2 activation can collaborate with the signaling of diverse PRRs to up-regulate IL-1β and IL-18 production in monocytic cells.

**Figure 2:**
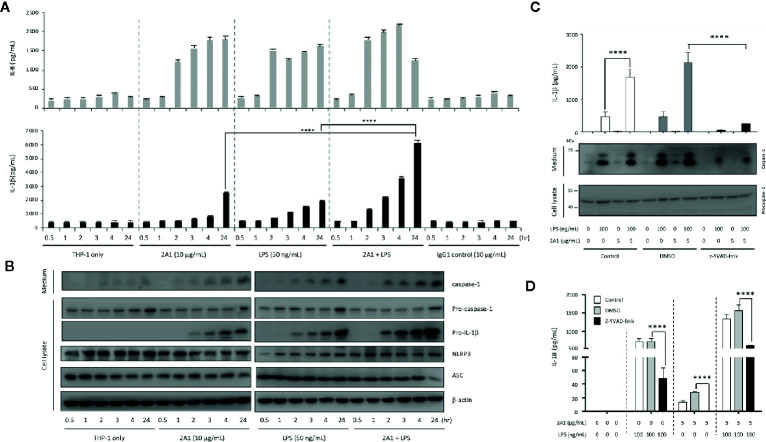
Caspase-1 activity is required for EMR2-induced IL-1β and IL-18 production in THP-1 cells. **(A)** ELISA analyses of IL-1β and IL-8 in the supernatants of THP-1 cells cultured on plates coated without or with 2A1 mAb in the absence or presence of LPS for different periods as indicated. (n=3, mean ± SEM; one-way ANOVA. **(B)** WB analyses of relevant protein components of the NLRP3 inflammasome in cell supernatant and cell lysate of THP-1 cells as treated in **(A)**. **(C, D)** The levels of IL-1β **(C)** and IL-18 **(D)** in the supernatants of THP-1 cells pre-treated with or without z-YVAD-fmk (50 μM), followed by stimulation with 2A1 mAb and LPS as indicated. (n=3, mean ± SEM; one-way ANOVA. The bottom panel in **(C)** showed the WB analyses of active capase-1 and pro-capase-1 in the cell supernatant and cell lysate, respectively. ****p<0.0001.

Along with the time-dependent IL-1β up-regulation, increased levels of pro-IL-1β and active caspase-1 were detected in the cell lysate and conditioned medium, respectively, of cells co-treated with LPS and 2A1 than those treated individually with 2A1 or LPS (**Figures 2A, B**). Conversely, EMR2-induced IL-8 levels remained unchanged in the presence of LPS, indicating a specific synergistic effect of EMR2 activation on IL-1β up-regulation (**Figure 2A**). Critically, EMR2-induced IL-1β and IL-18 up-regulation was markedly diminished in cells pre-treated with an irreversible caspase-1 inhibitor, z-YVAD-fmk, confirming the essential role of active caspase-1 in EMR2-promoted IL-1β and IL-18 production (**Figures 2C, D**). Taken together, activation of EMR2 in THP-1 cells by immobilized, but not soluble, 2A1 mAb significantly up-regulates IL-1β and IL-18 production in the presence of PAMPs, most likely due to inflammasome activation.

### EMR2 Stimulation Activates the NLRP3 Inflammasome

**Figure 3:**
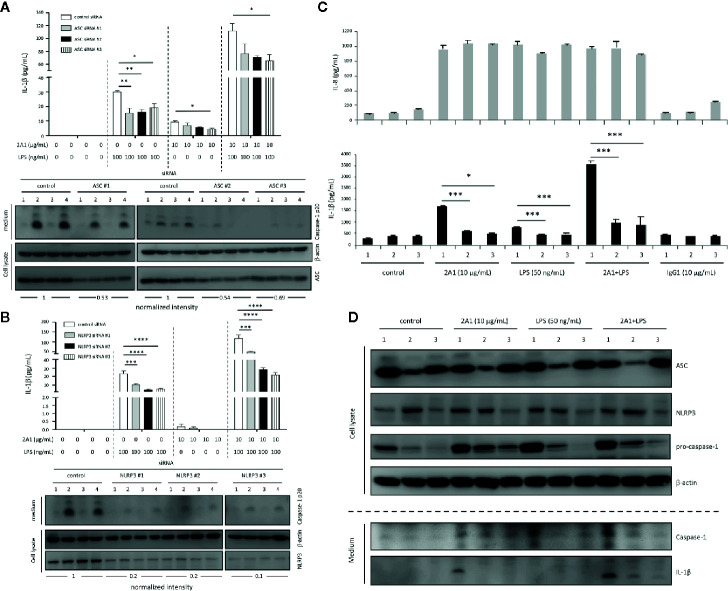
EMR2-enhanced IL-1β production in THP-1 cells is ASC- and NLRP3-dependent. **(A, B)** THP-1 cells were transfected with the ASC-specific siRNAs **(A)** or NLRP3-specific siRNAs **(B)** then cultured on plates coated without or with 2A1 mAb in the absence or presence of LPS for 24 h. A scrambled siRNA was included as a negative control. IL-1β in supernatant was analyzed by ELISA (top panel), while active caspase-1, ASC and NLRP3 protein expression was verified by WB analyses as indicated (bottom panel). Treatment of cells included: lane 1, cells only; lane 2, LPS alone; lane 3, immobilized 2A1 alone; lane 4, immobilized 2A1 plus LPS. The siRNA knock down efficiency was shown as a ratio determined by quantitative comparison of the density of the ASC and NLRP3 protein bands normalized to the β-actin band. **(C, D)** The parental (# 1), def-ASC (# 2), and def-NLRP3 (# 3) THP-1 cell lines were cultured on plates coated without or with 2A1 mAb in the absence or presence of LPS for 24 h. Cell supernatant was collected and analyzed for IL-8 and IL-1β by ELISA **(C)**. The expression of relevant proteins (NLRP3, ASC, caspase-1, procaspase-1, IL-1β) in the supernatant and cell lysate was examined by WB analyses as indicated **(D)**. Data are means ± SEM of at least three independent experiments performed in triplicate and analyzed by one-way ANOVA. ^*^ *p*<0.05, **p<0.01, ***p<0.001, ****p<0.0001 versus the THP-1 control group.

The NLRP3 inflammasome is the most common inflammasome activated by diverse PAMPs in monocytes and was reported to be elicited by FHR1-EMR2 interaction (25). We hence examined the role of 2A1-induced EMR2 ligation in NLRP3 inflammasome activation by first performing gene-specific siRNA knock down targeting ASC and NLRP3. As shown, up-regulated IL-1β production induced by the combined LPS and 2A1 treatment was greatly attenuated in THP-1 cells transfected with the ASC- or NLRP3-specific siRNAs, but not the scrambled control siRNA. Concomitantly, reduced levels of active caspase-1 were found in ASC- and NLRP3-knock down cells (**Figures 3A, B**).

Thereafter, we employed commercially available ASC-deficient (def-ASC) and NLRP3-deficient (def-NLRP3) THP-1 cell lines to clarify the role of EMR2-induced signaling in activating the NLRP3 inflammasome. Comparable EMR2 expression levels were detected in all three THP-1 cell lines, suggesting no direct roles for ASC and NLRP3 in regulating EMR2 expression (**Supplementary Figure 1D**). As expected, the enhanced IL-1β levels induced by 2A1 plus LPS in parental THP-1 cells were greatly diminished in def-ASC and def-NLRP3 cells (**Figure 3C**). Likewise, notably reduced levels of IL-1β and active caspase-1 were detected in the conditioned medium of def-ASC and def-NLRP3 cells (**Figure 3D**). Conversely, comparable IL-8 levels were induced in all three cell lines treated with 2A1, LPS, or 2A1 plus LPS, again supporting the specific role of ASC and NLRP3 in EMR2-promoted IL-1β production (**Figure 3C**). Collectively, these results establish a role for EMR2-mediated signaling in promoting NLRP3 inflammasome activation.

### EMR2 Ligation Triggers the Activation Signal for NLRP3 Inflammasome Activation

**Figure 4:**
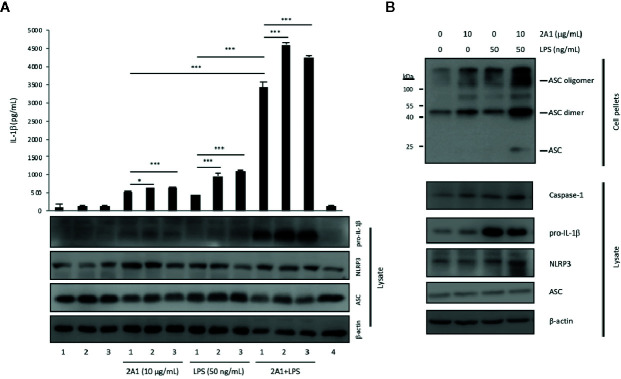
EMR2 ligation triggered the activation signal for NLRP3 inflammasome activation and induced ASC oligomerization in THP-1 cells. **(A)** ELISA analysis of IL-1β in the supernatants of THP-1 cells cultured on plates coated without or with 2A1 mAb in the absence or presence of LPS for 24 h. Cells were either treated without (lane 1) or with additional stimuli (lane 2, 5 mM ATP; lane 3, 100 mg/ml MSU), or 10 μg/ml mouse IgG_1_ (lane 4) as a negative control (n=3, mean ± SEM; one-way ANOVA. *p<0.05, ^***^ *p*<0.001). **(B)** THP-1 cells were cultured on plates coated without or with 2A1 mAb in the absence or presence of LPS for 24 h. The ASC oligomers and relevant components of NLRP3 inflammasome in the cell lysate and pellets were analyzed by Western blotting as indicated.

In accordance with the canonical two-signal activation mechanism of NLRP3 inflammasome (8, 9), our results suggested that 2A1-activated EMR2 most likely elicited the activation (2^nd^) signal. To validate this, we incubated THP-1 cells with 2A1 in the absence or presence of exogenous ATP or monosodium urate (MSU) crystal, two typical triggers of the activation signal. As reported elsewhere, ATP and MSU treatments lead to significant IL-1β up-regulation in the presence, but not in the absence, of LPS. However, no apparent IL-1β enhancement was detected in cells co-stimulated with 2A1 plus ATP or MSU versus cells treated with 2A1 alone (**Figure 4A**). Additionally, ASC oligomerization, a signature feature of NLRP3 inflammasome assembly and activation, was more readily identified in cells co-treated with LPS and 2A1 than those treated singly with LPS or 2A1 (**Figure 4B**, **Supplementary Figure 2**). These results indicate strongly that 2A1-induced EMR2 activation predominantly provides the activation signal for NLRP3 inflammasome activation.

**Figure 5:**
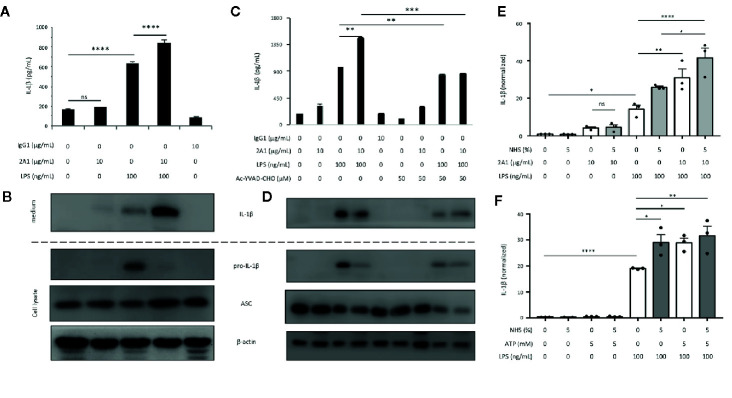
EMR2 ligation triggers the activation signal for NLRP3 inflammasome activation in human primary monocytes. **(A**–**F)** Isolated CD14^+^ primary monocytes (2 x 10^6^ cells/well) were incubated in 6-well plates coated without or with 2A1 mAb in the absence or presence of LPS for 24 h **(A**–**D)**. When necessary, cells were pretreated with Ac-YVAD-CHO (50 μM) for 1 h before treatment with LPS and 2A1 **(C, D)** or cultured in medium containing 5% NHS or ATP (5 mM) as described **(E, F)**. Cell supernatants were collected and analyzed for IL-1β by ELISA **(A, C, E, F)**. Cell lysates were analyzed by WB analysis for relevant proteins as indicated **(B, D)**. Data are means ± SEM of three independent experiments performed in triplicate using cells from three different donors **(E, F)**. Data were analyzed by one-way ANOVA. ^*^ *p*<0.05, ^**^ *p*<0.01, ^***^ *p*< 0.001, ^****^ *p*<0.0001 versus the control group. ns, non significant.

Unlike murine myeloid cells, human primary monocytes promptly secrete mature IL-1β in response to the PAMP-triggered priming signal alone. This is mainly due to the heightened responsiveness of human primary monocytes, leading to the release of endogenous ATP or activation of the “alternative” inflammasome activation pathway following PAMP stimulation (27–30). To attest to the role of EMR2 ligation in inducing the NLRP3 inflammasome activation signal, primary CD14^+^ monocytes were isolated and incubated with 2A1 in the absence or presence of LPS. In line with previous reports, significant IL-1β production was induced in cells receiving LPS treatment alone. Importantly, cells treated with LPS plus 2A1 produced markedly higher IL-1β levels, while those stimulated with 2A1 alone generated only basal IL-1β levels as did control cells (**Figures 5A–D**). Again, the enhanced IL-1β induced by LPS or LPS plus 2A1 in primary monocytes was significantly attenuated in the presence of a selective inhibitor of caspase-1, Ac-YVAD-CHO (**Figures 5C, D**).

During the course of this study, Irmscher *et al.* identified the complement system protein FHR1 as an EMR2-specific ligand and showed that FHR1-EMR2 interaction triggered NLRP3 inflammasome activation in primary monocytes (25). Intriguingly, the FHR1-induced NLRP3 inflammasome activation took place only in the presence of normal human serum (NHS) and when FHR1 was immobilized (25). These findings are somewhat similar to ours and prompted us to investigate the effect of NHS on 2A1-induced EMR2 activation. Interestingly, combined treatment of NHS and 2A1 generated little effect on IL-1β production in primary monocytes in comparison to cells treated with NHS or 2A1 alone. Nevertheless, NHS was efficient in up-regulating IL-1β production by monocytes treated with LPS or LPS plus 2A1 (**Figure 5E**). Likewise, addition of NHS, ATP, or both in the monocyte culture increased IL-1β levels significantly only in the presence of LPS (**Figure 5F**). Similar results were also observed in THP-1 cells subjected to the same culture conditions (**Supplementary Figure 3**). Hence, unlike in the case of FHR1, NHS is not required for 2A1-induced EMR2 activation. We conclude that the major role of 2A1-elicited EMR2 signaling is to trigger the activation (2^nd^) signal for NLRP3 inflammasome activation.

### EMR2-Induced NLRP3 Inflammasome Activation Signal Is Mediated *via* the PLC-β/Akt/Ca^2+^/MAPK/NF-κB Axes Downstream of Gα_16_

Our previous studies have established that 2A1-elicited EMR2 signaling activated the Gα_16_/PLC-β/PI3K/Akt/MAPK/NF-κB axes (24). On the other hand, FHR1-induced EMR2 activation involved a Gβγ-dependent PLC-sensitive pathway (25). Gα_16_ belongs to the Gα_q_ subfamily that is known to activate PLC-β isoforms, leading to the generation of diacylglycerol (DAG) and inositol triphosphate (IP_3_), which then triggered MAPK and NF-κB activation as well as intracellular calcium ion (Ca^2+^) mobilization, respectively (31). Similarly, the Gβγ subunits are also able to signal independently *via* PLC-β isoforms when dissociated from the active Gα subunit (32). To dissect the 2A1-induced EMR2 signaling cascades that triggered the NLRP3 inflammasome activation signal, we performed biochemical analyses using gene-specific siRNAs and diverse signaling inhibitors in THP-1 cells and monocytes.

**Figure 6:**
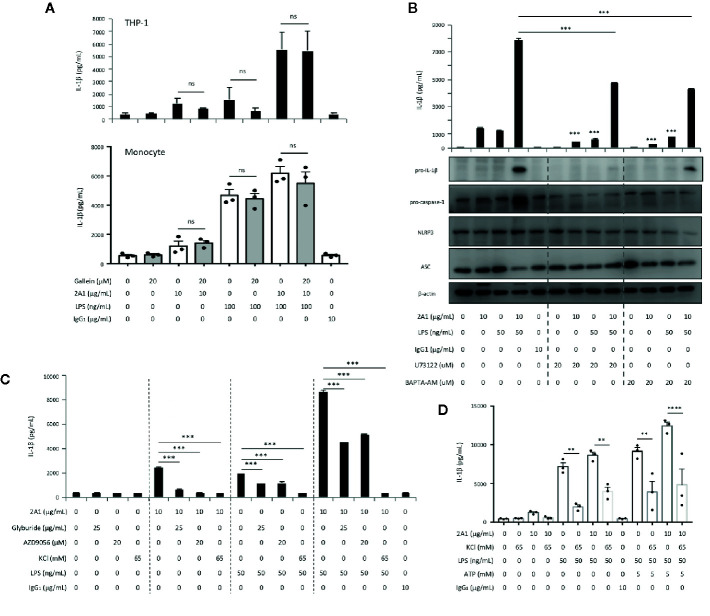
EMR2-mediated signaling in THP-1 cells induces a Gβγ-independent PLC-β activation and Ca^2+^ mobilization signaling activity, leading to K^+^ efflux and NLRP3 inflammasome activation. **(A, B)** Culture supernatants of THP-1 cells (**A** and **B** top panel) and monocytes (**A**, bottom panel) treated with indicated conditions for 24 h were collected for the detection of IL-1β by ELISA and relevant NLRP3 inflammasome proteins by western blotting analyses (**B**, bottom panel). **(C, D)** Culture supernatants of THP-1 cells **(C)** and monocytes **(D)** treated with indicated conditions for 24 h were collected for the detection of IL-1β by ELISA. Data are means ± SEM of at least three independent experiments performed in triplicate in THP-1 cells **(A–C)** or monocytes from three different donors **(A, D)**. Data were analyzed by one-way ANOVA. ns, non-significant, ^**^ *p*<0.01, ^***^ *p*< 0.001, ^****^ *p*<0.0001 versus the control group. Gallein, Gβγ inhibitor; U73122, PLC inhibitor; BAPTA-AM, Ca^2+^ chelator; Glyburide, inhibitor of the sulfonylurea receptor 1 (SUR1) subunit of the ATP-sensitive potassium channels; AZD9056, a selective P2X7 receptor antagonist; KCl, high extracellular KCl concentration served to inhibit cellular K^+^ efflux.

As expected, 2A1-enhanced IL-1β levels were reduced in THP-1 cells transfected with Gα_16_-specific siRNAs but not scrambled siRNA controls (**Supplementary Figure 4A**). Intriguingly, no significant effects on IL-1β production were observed by Gβγ inhibition both in THP-1 cells and monocytes, whereas the use of PLC inhibitor (U73122) and cell-permeable Ca^2+^ chelator (BAPTA-AM) attenuated 2A1-enhanced IL-1β release markedly (**Figures 6A, B**). In parallel, 2A1-induced IL-1β up-regulation was diminished following the blocking of cellular K^+^ efflux in THP-1 cells and monocytes (**Figures 6C, D**). These results indicate that 2A1-induced EMR2 activation in monocytes stimulates a Gα_16_-dependent (but Gβγ-independent) Ca^2+^ mobilization signaling activity leading to intracellular K^+^ efflux, which is generally considered a common trigger of the NLRP3 inflammasome activation signal (confirmed in **Supplementary Figure 4B**).

**Figure 7:**
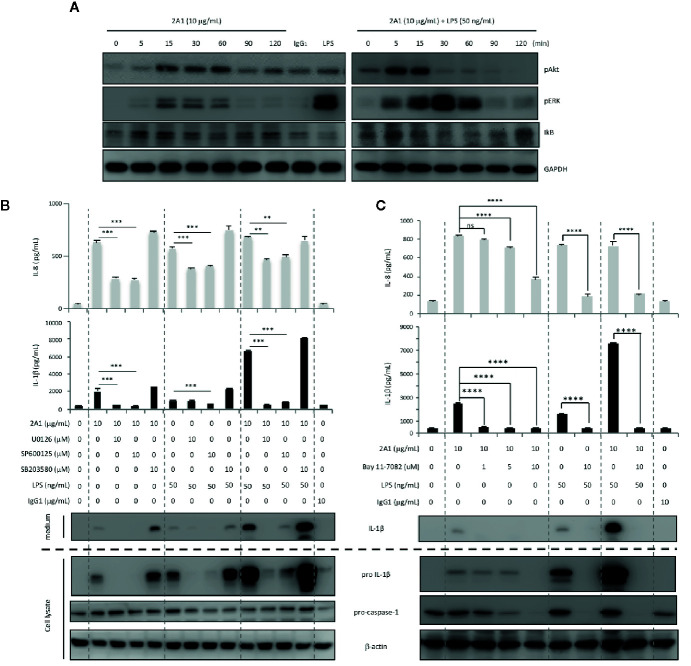
EMR2-mediated signaling in THP-1 cells results in phosphorylation and activation of Akt, ERK, and NF-κB, leading to NLRP3 inflammasome activation. **(A)** Western blot analyses of EMR2-mediated signaling in THP-1 cells incubated with 2A1 in the absence or presence of LPS at different time points as indicated. Cells treated with mouse IgG_1_ and LPS alone were included as negative and positive controls, respectively. Blots were probed to detect phospho-Akt, phospho-ERK, IκB and GAPDH level. **(B, C)** Culture supernatants of THP-1 cells treated with indicated conditions for 24 h were collected for the detection of IL-1β by ELISA (top panel) and relevant NLRP3 inflammasome proteins by western blotting analyses (bottom panel). Data are means ± SEM of at least three independent experiments performed in triplicate and analyzed by one-way ANOVA. ^**^ *p*<0.01, ^***^ *p*< 0.001, ^****^ *p*<0.0001 versus the control group. U0126, MEK1/2 inhibitor; SP600125, JNK inhibitor; SB203580, p38 inhibitor; Bay 11-7082, IκB kinase inhibitor.

**Figure 8:**
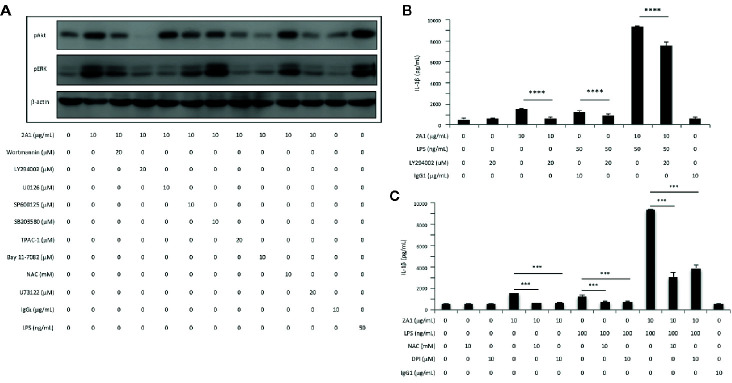
EMR2-mediated signaling in THP-1 cells induces Akt activation and ROS production, leading to NLRP3 inflammasome activation. **(A)** Western blotting analyses of EMR2-mediated signaling in THP-1 cells incubated with or without 2A1 and specific signaling inhibitors as indicated for 30 min. Blots were probed to detect phospho-Akt, phospho-ERK and β-actin level. Cells treated with mouse IgG_1_ and LPS were included as negative and positive controls, respectively. **(B, C)** Culture supernatants of THP-1 cells treated with indicated conditions for 24 h were collected for the detection of IL-1β by ELISA. Data are means ± SEM of at least three independent experiments performed in triplicate and analyzed by one-way ANOVA. ^***^ *p*< 0.001, ^****^ *p*<0.0001 versus the control group. LY294002, PI3K inhibitor; NAC (N-Acetyl-L-cysteine) and DPI (Diphenyleneiodonium), ROS inhibitors.

**Figure 9:**
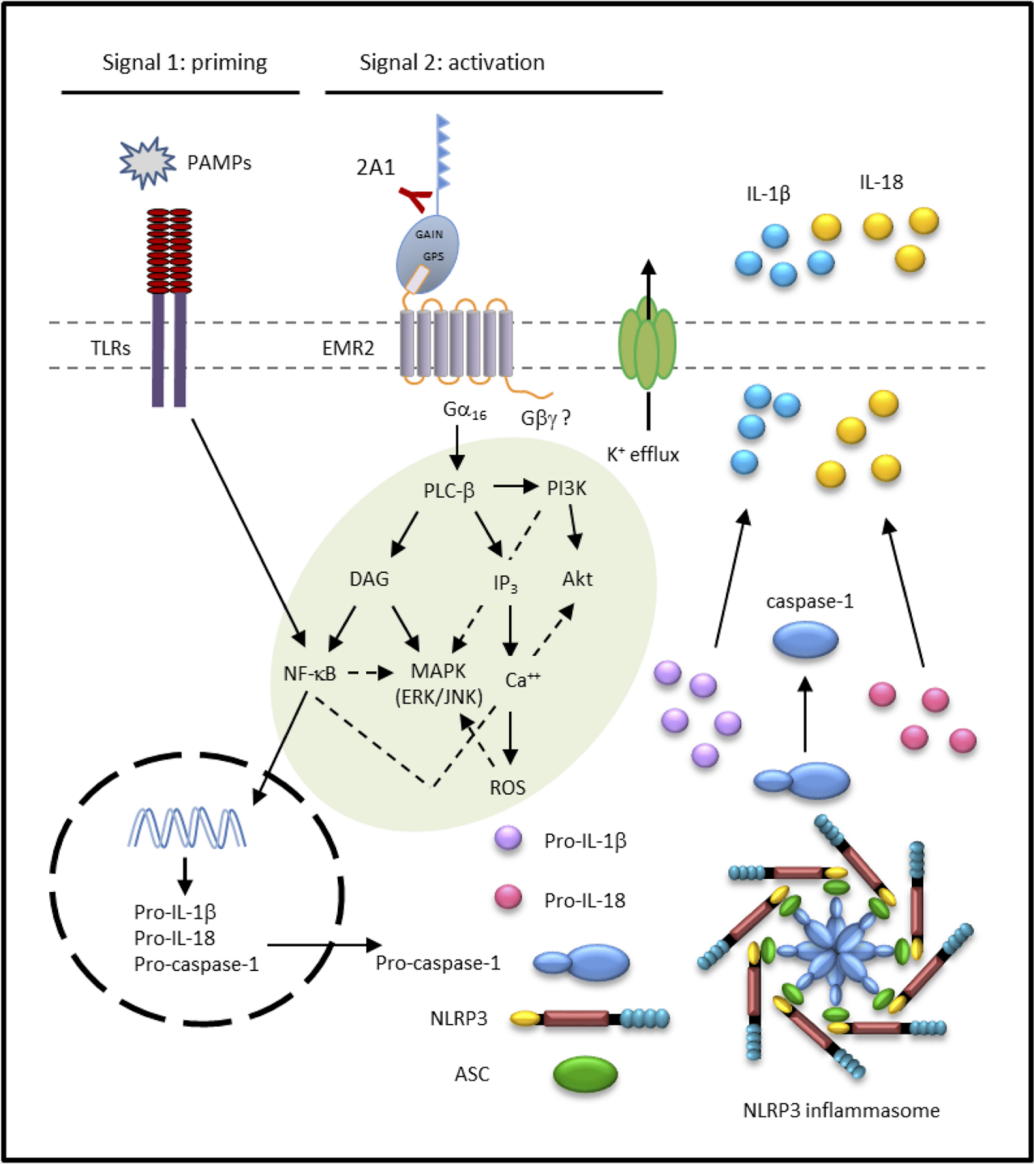
Proposed signaling pathways elicited by 2A1-activated EMR2 receptor that lead to NLRP3 inflammasome activation in monocytic cells. The solid lines indicate positive regulation and the dotted lines represent possible negative regulation/cross talk. ASC, Apoptosis-associated speck-like protein containing a CARD; DAG, diacylglycerol; EMR2, EGF-like module-containing mucin-like hormone receptor-like 2; GAIN, GPCR autoproteolysis-inducing; GPS, GPCR proteolysis site; IP_3_, inositol triphosphate; MAPK, mitogen-activated protein kinase; NLRP3, NOD-, LRR-, and pyrin domain-containing protein 3; PAMP, pathogen-associated molecular pattern; PI3K, phosphoinositide 3-kinase; PLC, phospholipase C; TLRs, toll-like receptors; ROS, reactive oxygen species.

In line with our earlier findings, western blot analyses revealed time-dependent phosphorylation and/or activation of Akt, ERK, and IκB following 2A1-induced EMR2 signaling (24). Critically, these signaling activities were further enhanced in cells co-treated with 2A1 and LPS (**Figure 7A** and **Supplementary Figure 5A**). Concomitantly, EMR2-induced IL-1β up-regulation was mitigated significantly in cells pre-treated with selective inhibitors of ERK (U0126), JNK (SP600125), and IκB kinase (IKK)(Bay 11-7082), but not the p38 inhibitor (SB203580) (**Figures 7B, C**). Similarly, IL-8 production induced by EMR2 activation was also reduced by the inhibition of ERK, JNK and IKK but not p38, reconfirming our earlier results (24). Further extensive western blot analyses of 2A1-activated THP-1 cells treated with various signaling inhibitors revealed intricate signaling regulation and potential cross-talk among selective signaling molecules. As such, inhibition of PLC-β (with U73122), PI3K (with Wortmannin and LY294002), and NF-κB activation (with TPCA-1 and Bay11-7082) did not only inactivate Akt, but also ERK phosphorylation. By contrast, inhibition of ERK (with U0126) had no impact on Akt phosphorylation, while inhibition of reactive oxygen species (ROS) production (with NAC) specifically inactivated JNK but not ERK (**Figure 8A**, **Supplementary Figure 5B**) Therefore, inhibition of Akt (with LY294002) and ROS (with NAC and DPI) both lead to diminished IL-1β and IL-8 production in 2A1-activated THP-1 cells (**Figures 8B, C** and **Supplementary Figure 5C**). These results prompted us to propose a Gα_16_-mediated PLC-β dependent signaling network evoked by 2A1-elicited EMR2 activation that worked collectively to induce K^+^ efflux and hence the NLRP3 inflammasome activation signal (**Figure 9**).

## Discussion

EMR2/ADGRE2 is a human myeloid-restricted aGPCR strongly associated with diverse inflammatory pathologies such as RA, SIRS, and VU (20, 33). However, EMR2-mediated signaling events and their functional significance remained to be fully elucidated. In this study, we have unraveled the signaling mechanisms of EMR2 in inducing NLRP3 inflammasome activation in monocytes. The conclusion that EMR2-mediated signaling triggers the activation (2^nd^) signal for NLRP3 inflammasome is reminiscent of other inflammasome-activating GPCRs such as the calcium sensing receptor (CaSR) and its closely-related GPRC6A protein (10). Our findings hence confirm and mark EMR2 as one of a limited number of GPCRs known to activate NLRP3 inflammasome in human myeloid cells.

EMR2 is a typical aGPCR that undergoes autoproteolytic processing at the extracellular GPCR proteolysis site (GPS) and is expressed as a bipartite complex containing the extracellular N-terminal fragment (NTF) and the seven-transmembrane (7TM) C-terminal fragment (CTF) (34, 35). One of the aGPCR activation mechanisms is the tethered-ligand activation model, in which an aGPCR is activated when the NTF dissociates from CTF following the binding of its specific extracellular ligand(s), possibly with the help of mechanical cues (36, 37). This allows the exposure of the most N-terminal region of the CTF, called the *Stachel* peptide, which then acts as a tethered ligand to interact and activate its own 7TM region. Indeed, the EMR2-pC492Y variant has been shown to function as a mechanosensor in mast cells of VU patients and is prone to shed its NTF in response to vibratory challenge (21). As such, it is of great interest to note the compulsory requirement of 2A1 and FHR1 immobilization for efficient EMR2 activation. These results strongly suggested that the multivalent ligation of the receptor molecule by fixed 2A1 and FHR1 ligands likely induced the shedding/dislocation of EMR2-NTF and the subsequent activation of EMR2-CTF in monocytes. Whether EMR2-NTF is shed and, if so, the extent of shedding upon the binding of EMR2 to immobilized 2A1 and FHR1, remain to be characterized.

It is noteworthy to mention that the EMR2-pC492Y variant on mast cells is activated by the 2A1 mAb as well as its specific glycosaminoglycan ligand, DS (21, 38). The role of glycosaminoglycans in modulating inflammasome activation and inflammatory responses in general has been highlighted by numerous studies (39). For example, the abnormal accumulation of cellular glycosaminoglycans in lysosomal storage disorders was strongly implicated in the pathogenic activation of NLRP3 inflammasome (40). The small proteoglycan, biglycan, was shown to function as a danger signal and induce NLRP3 inflammasome activation by interacting with the TLR2/4 and purinergic P2X4/P2X7 receptors (41). On the other hand, chondroitin sulfate was recently found to attenuate MSU-induced macrophage inflammatory activation *in vitro* (42). Considering the ubiquitous presence of DS in extracellular matrix and cell surface proteoglycans, the role of EMR2 in modulating NLRP3 inflammasome activation by monocytes in response to quantitative and qualitative changes of DS in tissue microenvironments will be of great interest for a later study.

The identification of a Gα_16_-dependent PLC-β activation pathway induced by 2A1-activated EMR2 is consistent with our earlier findings (24). Importantly, EMR2 coupling to Gα_16_ and PLC-β activation was also demonstrated recently by Bhudia *et al.* who showed that HEK-293T cells co-expressing Gα_16_ and EMR2-CTF resulted in inositol monophosphate (IP_1_) accumulation as well as a strong increase of the NFAT-luciferase activity, two critical indicators of the activated Gα_16_/PLC-β/Ca^2+^ signaling axis (43). While these results reiterate the specific and strong coupling of EMR2 with the Gα_16_ protein, we failed to find the involvement of Gβγ in 2A1-induced EMR2 signaling, unlike that of FHR1-EMR2 interaction. Interestingly, a recent study by Naranjo *et al.* similarly revealed that vibratory interaction of DS and EMR2-pC492Y in mast cells induced a Gβγ-, Gα_q/11_-, and Gα_i/o_-independent mechanism leading to specific activation of PLC-β, PI3K, ERK1/2 and a transient cytosolic calcium increase (38). One possibility for these differential signaling activities might be due to the potential biased signaling mediated by EMR2 in different cell types (monocyte vs. mast cell) in response to different stimuli (2A1 vs. FHR1 plus NHS vs. DS and vibration). This possibility is further supported by the fact that we did not find a role for NHS in 2A1-induced EMR2 activation (**Figure 5**), while it is absolutely required for EMR2 activation elicited by FHR1. The exact role of NHS in FHR1-induced NLRP3 inflammasome activation warrants further investigation.

Although most of the downstream signaling effectors of Gα_16_/PLC-β activation, including ERK1/2, JNK, NF-κB, and Akt have been reported in our previous study and re-confirmed here and elsewhere, we further showed that Ca^2+^ mobilization, ROS production, and K^+^ efflux are critically involved in the signaling pathways of EMR2-induced NLRP3 inflammasome activation (**Figure 9**). While both Ca^2+^ and ROS are well-known triggers of NLRP3 inflammasome, cellular K^+^ efflux has been considered to be the universal inducer of NLRP3 inflammasome activation, involved in the signaling of almost all NLRP3 stimuli, such as ATP, nigericin, and particulate matter (6, 8). Hence, we conclude that 2A1-induced EMR2 activation is initiated by Gα_16_ coupling and PLC-β stimulation, followed by the activation of Akt, ERK1/2, JNK, NFkB, Ca^2+^ mobilization, and ROS production, likely *via* the generation/activation of DIG, IP_3_, and PI3K. The collective actions of these signaling events eventually trigger K^+^ efflux and NLRP3 inflammasome activation (**Figure 9**). It is of great interest to note that PLC-β, Akt, ERK1/2, NFkB, and Ca^2+^ mobilization are consistently identified in several independent studies of EMR2 activation and signaling. These signaling intermediates along with EMR2 itself hence represent potential targets of therapeutic intervention for relevant inflammatory disorders.

## Data Availability Statement

The original contributions presented in the study are included in the article/**Supplementary Material**. Further inquiries can be directed to the corresponding author.

## Author Contributions

K-YI, W-YT, W-CW, and H-HL performed experiments. K-YI, W-YT, SG, K-FN, and H-HL designed the experiments, analyzed, and interpreted the data. K-YI, W-YT, and H-HL wrote the manuscript. All authors contributed to the article and approved the submitted version.

## Funding

This study was supported by grants from the Chang Gung Memorial Hospital to W-YT (CMRPG2J0051 and CMRPG2K0261), and to H-HL (CMRPD1G0443, CMRPD1K0131, CMRPD1K0211, CMRPD1K0221, and CMRPD1K0301), and from the Ministry of Science and Technology (MOST), Taiwan to W-YT (MOST-108-2628-B-182A-005), and to H-HL (MOST-107-2320-B-182-006).

## Conflict of Interest

The authors declare that the research was conducted in the absence of any commercial or financial relationships that could be construed as a potential conflict of interest.

## Abbreviations

AAV, anti-neutrophil cytoplasmic antibody-associated vasculitis; aGPCR, adhesion GPCR; ASC, apoptosis-associated speck-like protein containing a CARD; DAG, diacylglycerol; DAMP, damage-associated molecular pattern; DC, dendritic cell; EGF, epidermal growth factor; DS, dermatan sulphate; EMR2, EGF-like module-containing mucin-like hormone receptor-like 2; FHR1, complement Factor H-related protein 1; GAIN, GPCR autoproteolysis-inducing; GPCR, G protein-coupled receptor; GPS, GPCR proteolysis site; IP_3_, inositol triphosphate; LPS, lipopolysaccharide; mAb, monoclonal antibody; MAPK, mitogen-activated protein kinase; MSU, monosodium urate; Mφ, macrophage; NHS, normal human serum; NLRP3, NOD-, LRR-, and pyrin domain-containing protein 3; Nφ, neutrophil; PAMP, pathogen-associated molecular pattern; PI3K, phosphoinositide 3-kinase; PLC, phospholipase C; PRR, pattern-recognition receptor; RA, rheumatoid arthritis; ROS, reactive oxygen species; SIRS, systemic inflammatory response syndrome; VU, vibratory urticaria.
