# Supplementary material for: Stimulation of Vibratory Urticaria-Associated Adhesion-GPCR, EMR2/ADGRE2, Triggers the NLRP3 Inflammasome Activation Signal in Human Monocytes
Source: Front Immunol. 2021 Jan 8;11:602016. doi: 10.3389/fimmu.2020.602016 (PMC7820815; doi:10.3389/fimmu.2020.602016)
Supplement: Supplementary file 2 [file Presentation_1.pptx]

## Slide 1
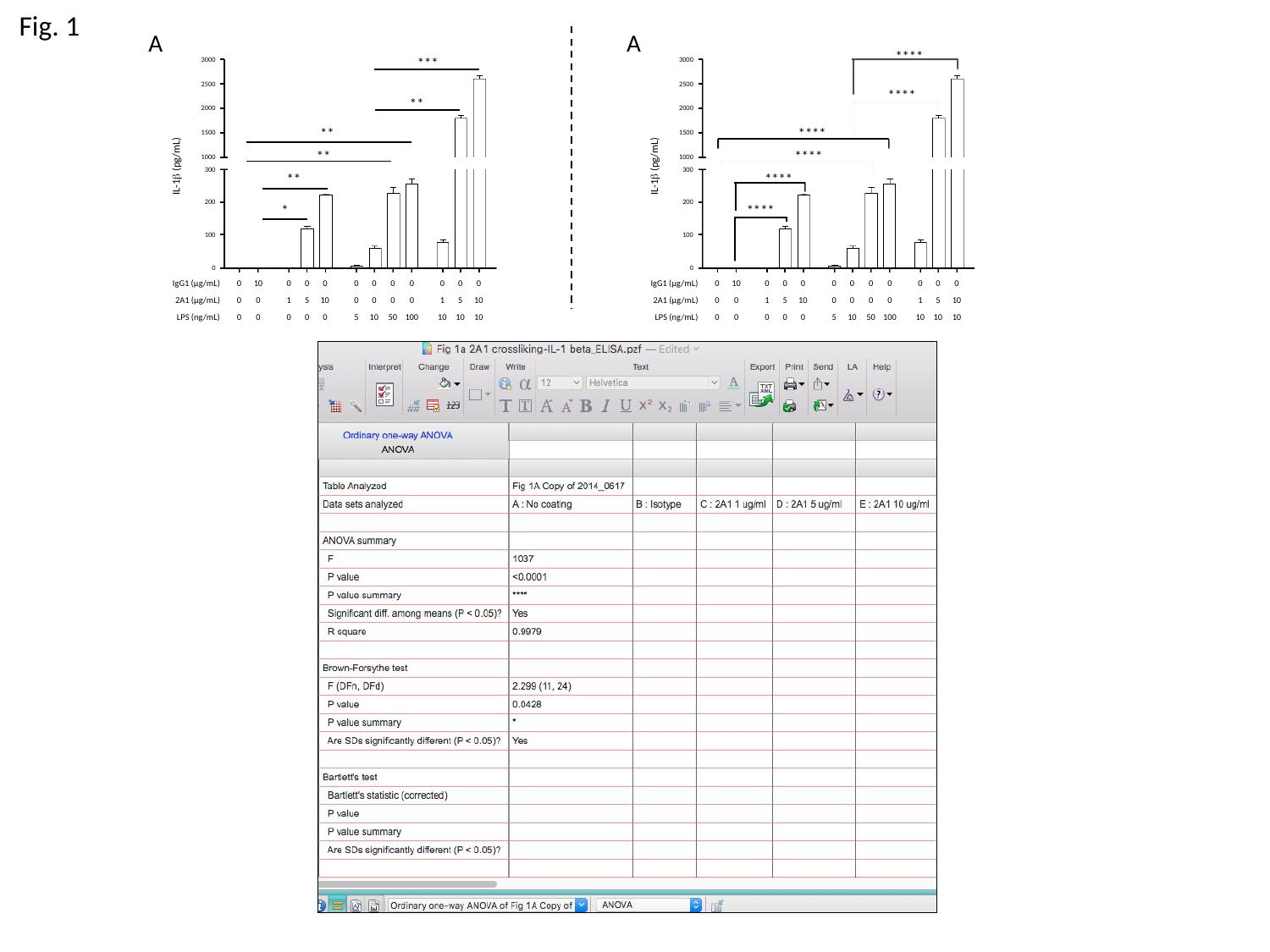

Fig. 1
A
A
****
3000
2500
****
2000
****
1500
****
1000
300
****
200
****
100
0
0
10
0
0
0
0
0
0
0
0
0
0
0
0
1
5
10
0
0
0
0
1
5
10
IL-1β (pg/mL)
IgG1 (μg/mL)
2A1 (μg/mL)
LPS (ng/mL)
0
0
0
0
0
5
10
50
100
10
10
10
***
3000
2500
**
2000
**
1500
**
1000
IL-1β (pg/mL)
300
**
200
*
100
0
IgG1 (μg/mL)
0
10
0
0
0
0
0
0
0
0
0
0
2A1 (μg/mL)
0
0
1
5
10
0
0
0
0
1
5
10
LPS (ng/mL)
0
0
0
0
0
5
10
50
100
10
10
10

## Slide 2
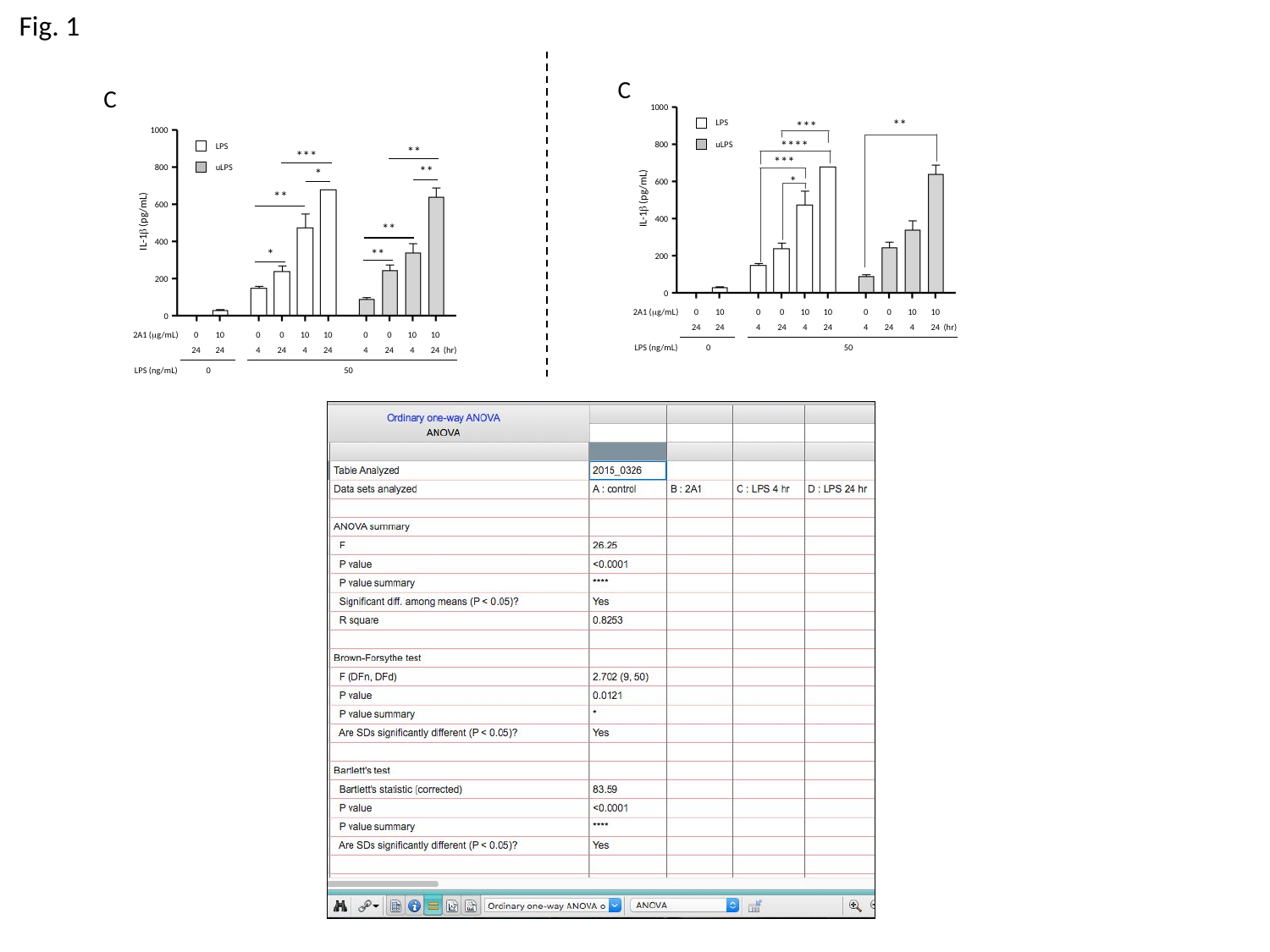

Fig. 1
C
1000
**
LPS
***
****
800
uLPS
***
*
600
IL-1β (pg/mL)
400
200
0
2A1 (μg/mL)
0
10
0
0
10
10
0
0
10
10
24
24
4
24
4
24
4
24
4
24 (hr)
LPS (ng/mL)
0
50
C
1000
LPS
**
***
800
uLPS
**
*
**
600
IL-1β (pg/mL)
**
400
**
*
200
0
2A1 (μg/mL)
0
10
0
0
10
10
0
0
10
10
24
24
4
24
4
24
4
24
4
24 (hr)
LPS (ng/mL)
0
50

## Slide 3
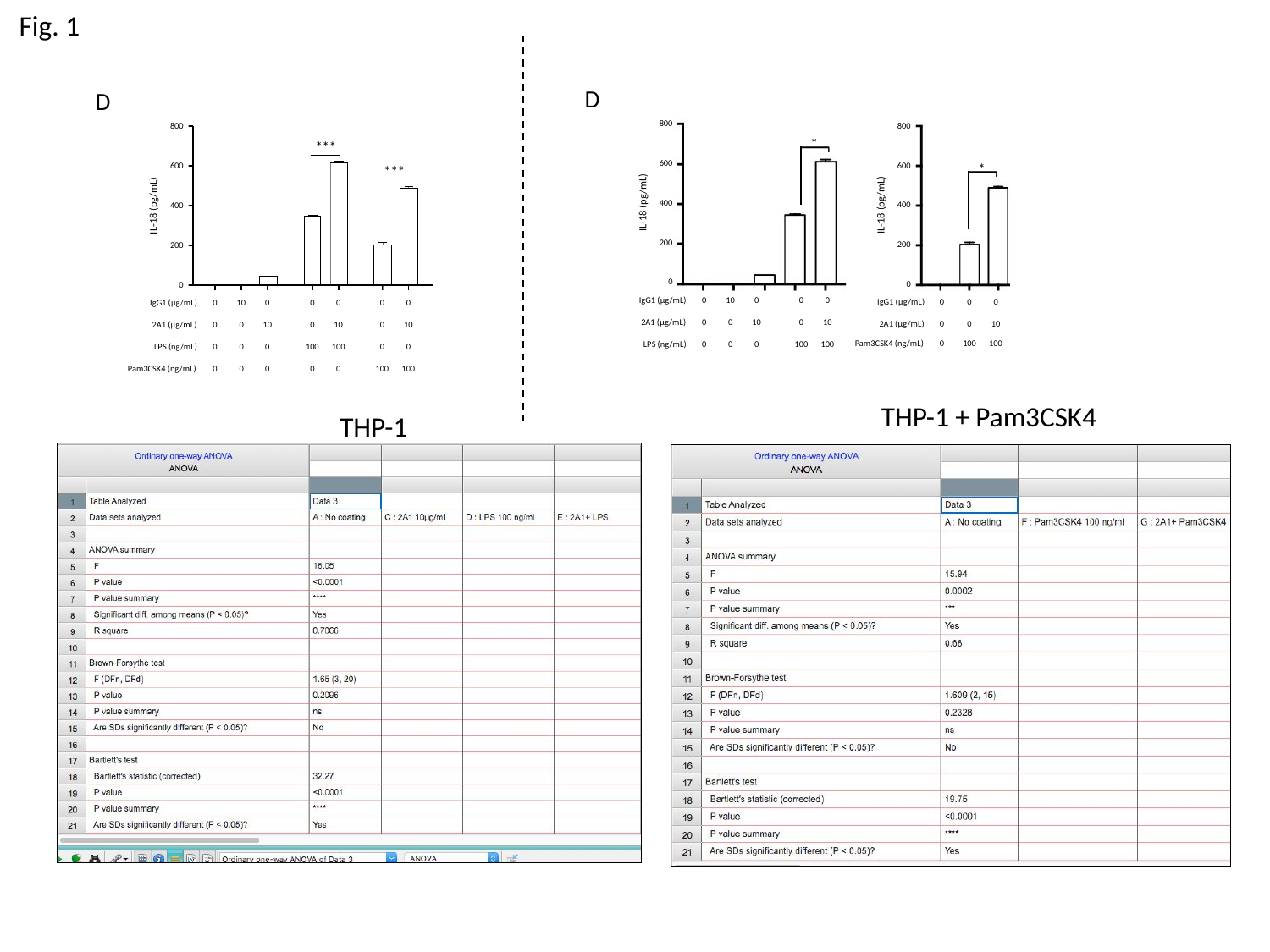

Fig. 1
D
800
600
IL-18 (pg/mL)
400
200
0
IgG1 (μg/mL)
0
10
0
0
0
2A1 (μg/mL)
0
0
10
0
10
LPS (ng/mL)
0
0
0
100
100
800
600
IL-18 (pg/mL)
400
200
0
*
0
IgG1 (μg/mL)
0
0
0
2A1 (μg/mL)
0
10
0
Pam3CSK4 (ng/mL)
100
100
*
D
800
600
IL-18 (pg/mL)
400
200
0
IgG1 (μg/mL)
0
10
0
0
0
0
0
2A1 (μg/mL)
0
0
10
0
10
0
10
LPS (ng/mL)
0
0
0
100
100
0
0
Pam3CSK4 (ng/mL)
0
0
0
0
0
100
100
***
***
THP-1 + Pam3CSK4
THP-1

## Slide 4
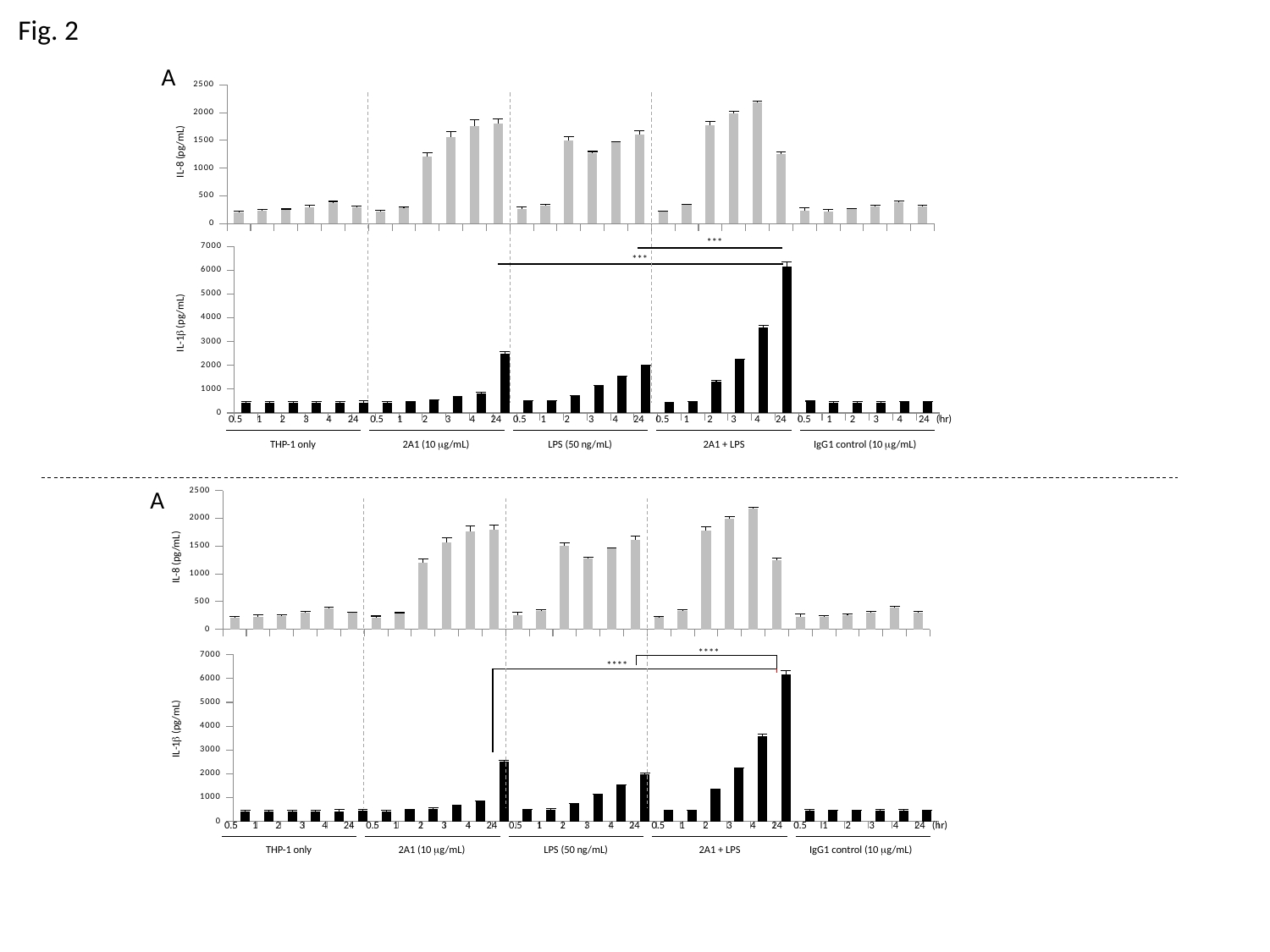

Fig. 2
A
### Chart
| Category | |
|---|---|
| 0.5hr | 207.0 |
| 1hr | 227.0 |
| 2hr | 242.0 |
| 3hr | 294.5 |
| 4hr | 372.0 |
| 24hr | 294.5 |
| 2A1 0.5hr | 214.5 |
| 2A1 1hr | 287.0 |
| 2A1 2hr | 1207.0 |
| 2A1 3hr | 1564.5 |
| 2A1 4hr | 1762.0 |
| 2A1 24hr | 1802.0 |
| LPS 0.5hr | 259.5 |
| LPS 1hr | 327.0 |
| LPS 2hr | 1499.5 |
| LPS 3hr | 1272.0 |
| LPS 4hr | 1472.0 |
| LPS 24hr | 1612.0 |
| 2A1+LPS 0.5hr | 222.0 |
| 2A1+LPS 1hr | 337.0 |
| 2A1+LPS 2hr | 1779.5 |
| 2A1+LPS 3hr | 1989.5 |
| 2A1+LPS 4hr | 2179.5 |
| 2A1+LPS 24hr | 1247.0 |
| IgG 0.5hr | 232.0 |
| IgG 1hr | 224.5 |
| IgG 2hr | 259.5 |
| IgG 3hr | 302.0 |
| IgG 4hr | 387.0 |
| IgG 24hr | 307.0 |IL-8 (pg/mL)
***
### Chart
| Category | |
|---|---|
| 0.5hr | 411.9999999999994 |
| 1hr | 416.9999999999994 |
| 2hr | 421.9999999999994 |
| 3hr | 416.9999999999994 |
| 4hr | 426.9999999999994 |
| 24hr | 437.0 |
| 2A1 0.5hr | 421.9999999999994 |
| 2A1 1hr | 467.0 |
| 2A1 2hr | 527.0 |
| 2A1 3hr | 661.9999999999999 |
| 2A1 4hr | 831.9999999999999 |
| 2A1 24hr | 2497.0 |
| LPS 0.5hr | 492.0 |
| LPS 1hr | 487.0 |
| LPS 2hr | 717.0 |
| LPS 3hr | 1142.0 |
| LPS 4hr | 1532.0 |
| LPS 24hr | 1972.0 |
| 2A1+LPS 0.5hr | 446.9999999999994 |
| 2A1+LPS 1hr | 471.9999999999994 |
| 2A1+LPS 2hr | 1322.0 |
| 2A1+LPS 3hr | 2222.0 |
| 2A1+LPS 4hr | 3577.0 |
| 2A1+LPS 24hr | 6157.0 |
| IgG 0.5hr | 447.0 |
| IgG 1hr | 432.0 |
| IgG 2hr | 437.0 |
| IgG 3hr | 437.0 |
| IgG 4hr | 457.0 |
| IgG 24hr | 447.0 |***
IL-1β (pg/mL)
0.5
1
2
3
4
24
0.5
1
2
3
4
24
0.5
1
2
3
4
24
0.5
1
2
3
4
THP-1 only
2A1 (10 μg/mL)
LPS (50 ng/mL)
2A1 + LPS
24
0.5
1
2
3
4
24 (hr)
IgG1 control (10 μg/mL)
A
### Chart
| Category | |
|---|---|
| 0.5hr | 207.0 |
| 1hr | 227.0 |
| 2hr | 242.0 |
| 3hr | 294.5 |
| 4hr | 372.0 |
| 24hr | 294.5 |
| 2A1 0.5hr | 214.5 |
| 2A1 1hr | 287.0 |
| 2A1 2hr | 1207.0 |
| 2A1 3hr | 1564.5 |
| 2A1 4hr | 1762.0 |
| 2A1 24hr | 1802.0 |
| LPS 0.5hr | 259.5 |
| LPS 1hr | 327.0 |
| LPS 2hr | 1499.5 |
| LPS 3hr | 1272.0 |
| LPS 4hr | 1472.0 |
| LPS 24hr | 1612.0 |
| 2A1+LPS 0.5hr | 222.0 |
| 2A1+LPS 1hr | 337.0 |
| 2A1+LPS 2hr | 1779.5 |
| 2A1+LPS 3hr | 1989.5 |
| 2A1+LPS 4hr | 2179.5 |
| 2A1+LPS 24hr | 1247.0 |
| IgG 0.5hr | 232.0 |
| IgG 1hr | 224.5 |
| IgG 2hr | 259.5 |
| IgG 3hr | 302.0 |
| IgG 4hr | 387.0 |
| IgG 24hr | 307.0 |IL-8 (pg/mL)
****
### Chart
| Category | |
|---|---|
| 0.5hr | 411.9999999999994 |
| 1hr | 416.9999999999994 |
| 2hr | 421.9999999999994 |
| 3hr | 416.9999999999994 |
| 4hr | 426.9999999999994 |
| 24hr | 437.0 |
| 2A1 0.5hr | 421.9999999999994 |
| 2A1 1hr | 467.0 |
| 2A1 2hr | 527.0 |
| 2A1 3hr | 661.9999999999999 |
| 2A1 4hr | 831.9999999999999 |
| 2A1 24hr | 2497.0 |
| LPS 0.5hr | 492.0 |
| LPS 1hr | 487.0 |
| LPS 2hr | 717.0 |
| LPS 3hr | 1142.0 |
| LPS 4hr | 1532.0 |
| LPS 24hr | 1972.0 |
| 2A1+LPS 0.5hr | 446.9999999999994 |
| 2A1+LPS 1hr | 471.9999999999994 |
| 2A1+LPS 2hr | 1322.0 |
| 2A1+LPS 3hr | 2222.0 |
| 2A1+LPS 4hr | 3577.0 |
| 2A1+LPS 24hr | 6157.0 |
| IgG 0.5hr | 447.0 |
| IgG 1hr | 432.0 |
| IgG 2hr | 437.0 |
| IgG 3hr | 437.0 |
| IgG 4hr | 457.0 |
| IgG 24hr | 447.0 |****
IL-1β (pg/mL)
0.5
1
2
3
4
24
0.5
1
2
3
4
24
0.5
1
2
3
4
24
0.5
1
2
3
4
THP-1 only
2A1 (10 μg/mL)
LPS (50 ng/mL)
2A1 + LPS
24
0.5
1
2
3
4
24 (hr)
IgG1 control (10 μg/mL)

## Slide 5
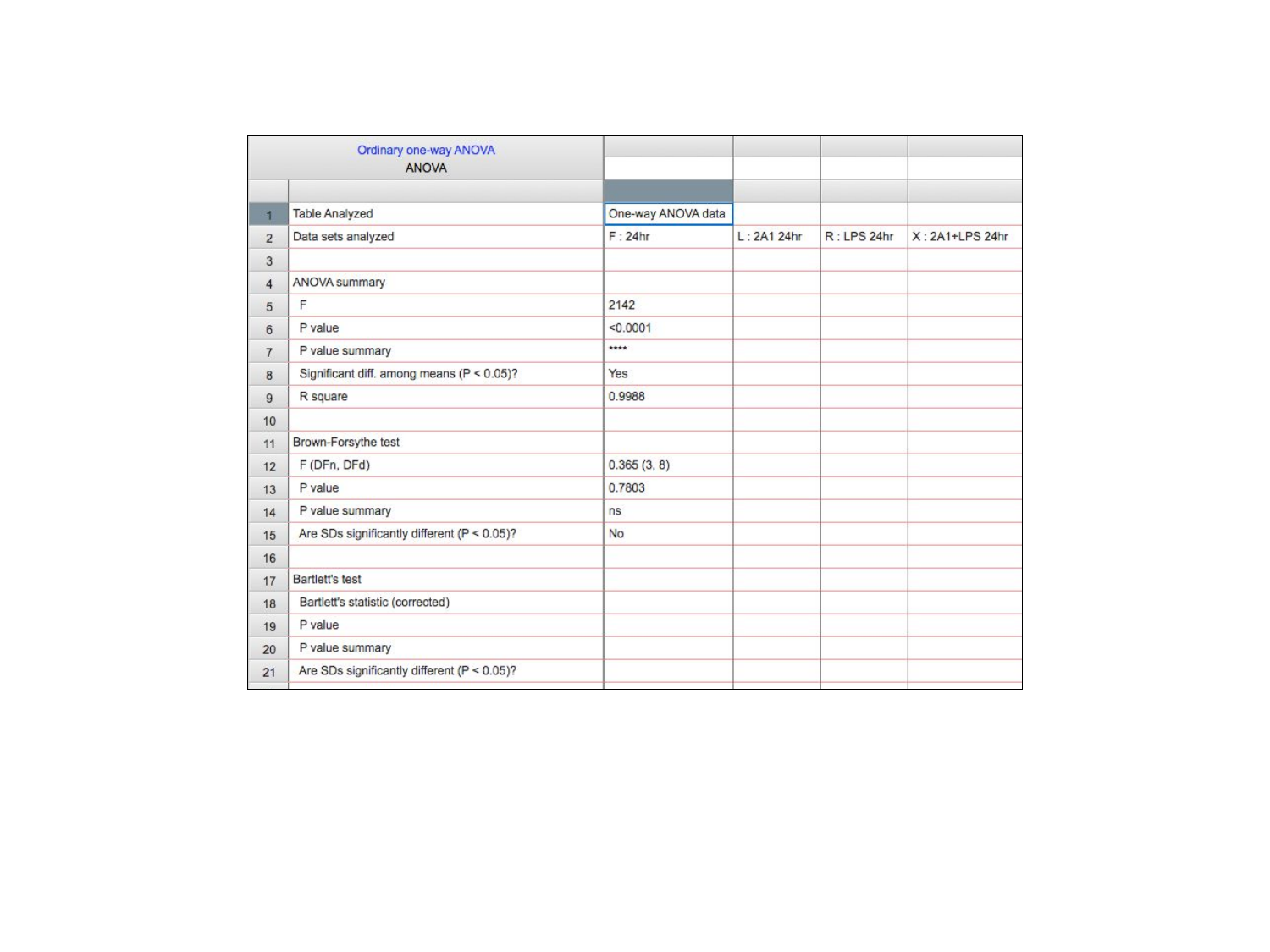

## Slide 6
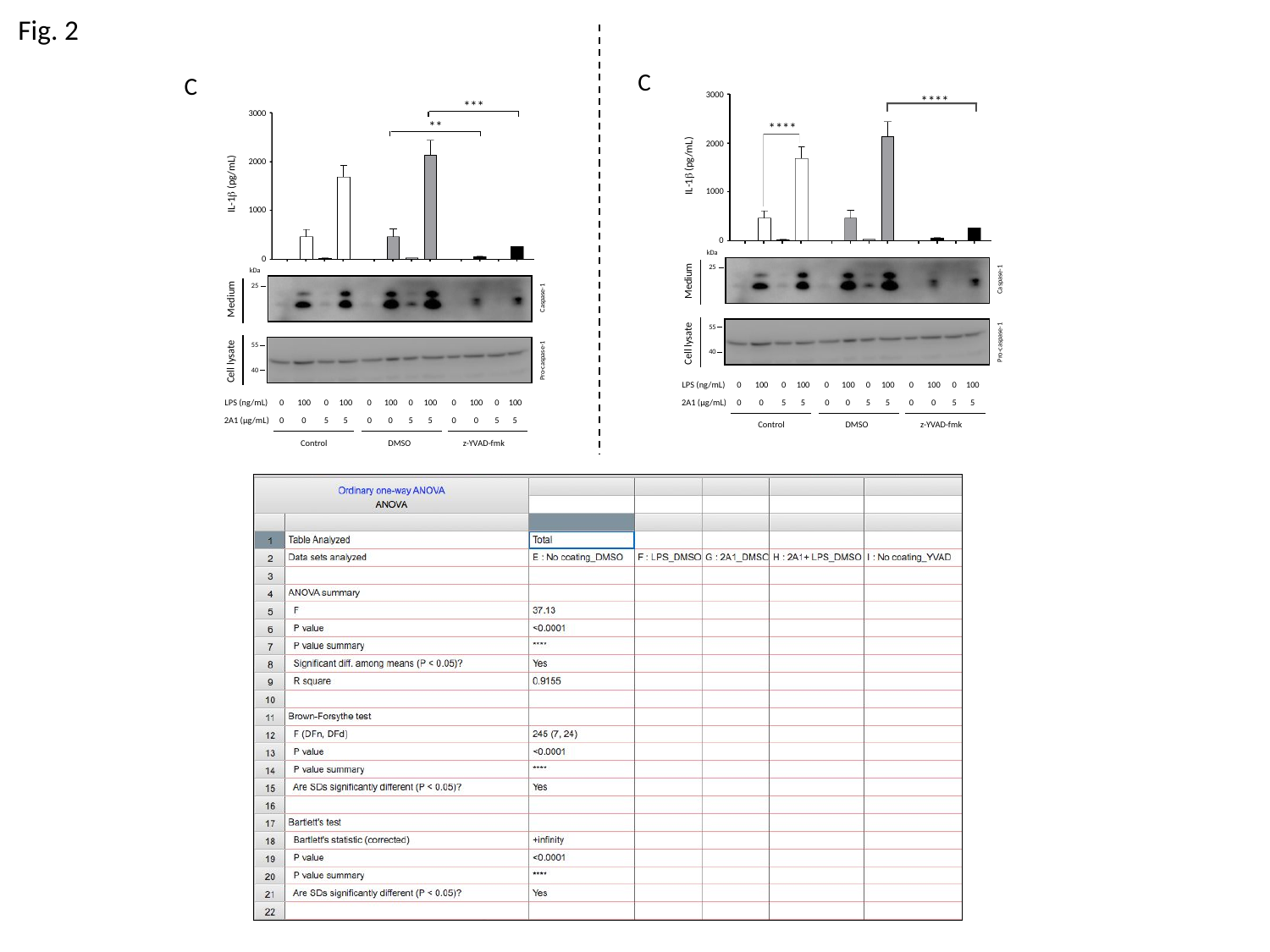

Fig. 2
C
C
3000
****
****
2000
IL-1β (pg/mL)
1000
0
kDa
25
Medium
Caspase-1
55
Cell lysate
Pro-caspase-1
40
LPS (ng/mL)
0
100
0
100
0
100
0
100
0
100
0
100
2A1 (μg/mL)
0
0
5
5
0
0
5
5
0
0
5
5
Control
DMSO
z-YVAD-fmk
***
3000
**
2000
IL-1β (pg/mL)
1000
0
kDa
25
Medium
Caspase-1
55
Cell lysate
Pro-caspase-1
40
LPS (ng/mL)
0
100
0
100
0
100
0
100
0
100
0
100
2A1 (μg/mL)
0
0
5
5
0
0
5
5
0
0
5
5
Control
DMSO
z-YVAD-fmk

## Slide 7
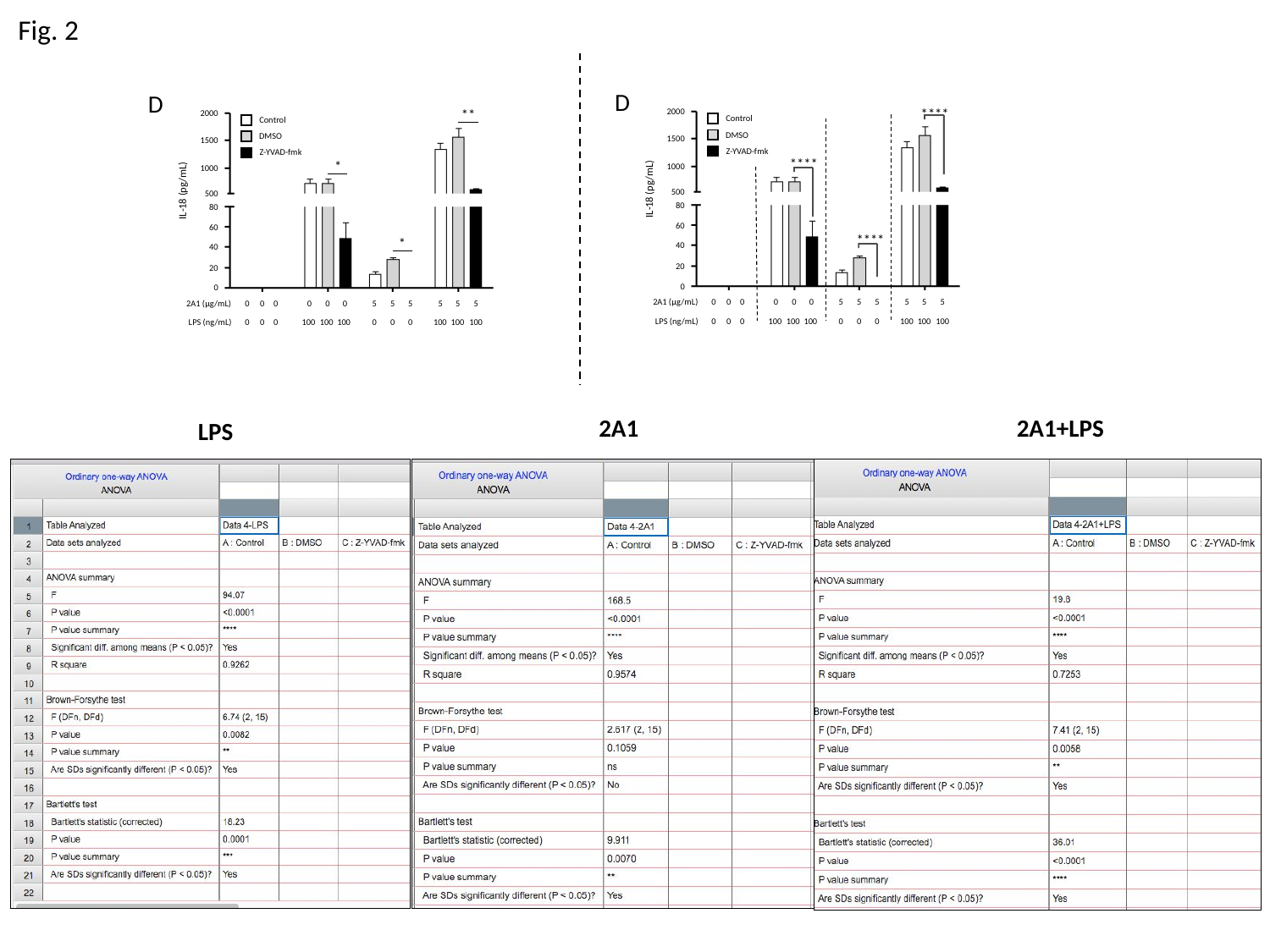

Fig. 2
D
D
****
2000
Control
DMSO
Z-YVAD-fmk
1500
****
1000
IL-18 (pg/mL)
500
80
60
****
40
20
0
2A1 (μg/mL)
0
0
0
0
0
0
5
5
5
5
5
5
LPS (ng/mL)
0
0
0
100
100
100
0
0
0
100
100
100
**
2000
Control
DMSO
Z-YVAD-fmk
1500
*
1000
IL-18 (pg/mL)
500
80
60
*
40
20
0
2A1 (μg/mL)
0
0
0
0
0
0
5
5
5
5
5
5
LPS (ng/mL)
0
0
0
100
100
100
0
0
0
100
100
100
2A1+LPS
2A1
LPS

## Slide 8
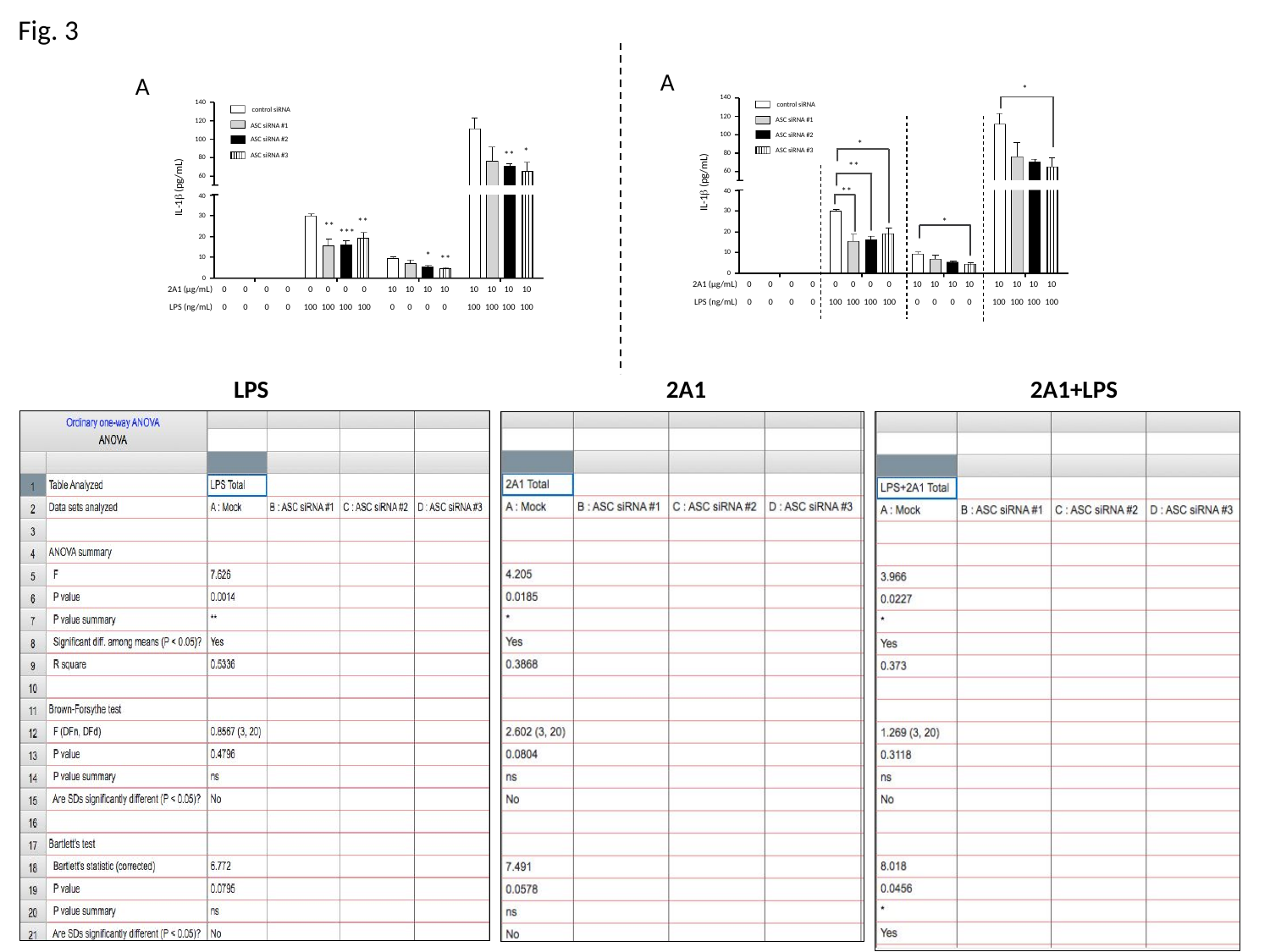

Fig. 3
A
*
140
control siRNA
ASC siRNA #1
ASC siRNA #2
ASC siRNA #3
120
100
*
80
**
60
IL-1β (pg/mL)
**
40
30
*
20
10
0
2A1 (μg/mL)
0
0
0
0
0
0
0
0
10
10
10
10
10
10
10
10
LPS (ng/mL)
0
0
0
0
100
100
100
100
0
0
0
0
100
100
100
100
A
140
control siRNA
ASC siRNA #1
ASC siRNA #2
ASC siRNA #3
120
100
*
**
80
60
IL-1β (pg/mL)
40
30
**
**
***
20
*
10
**
0
2A1 (μg/mL)
0
0
0
0
0
0
0
0
10
10
10
10
10
10
10
10
LPS (ng/mL)
0
0
0
0
100
100
100
100
0
0
0
0
100
100
100
100
LPS
2A1
2A1+LPS

## Slide 9
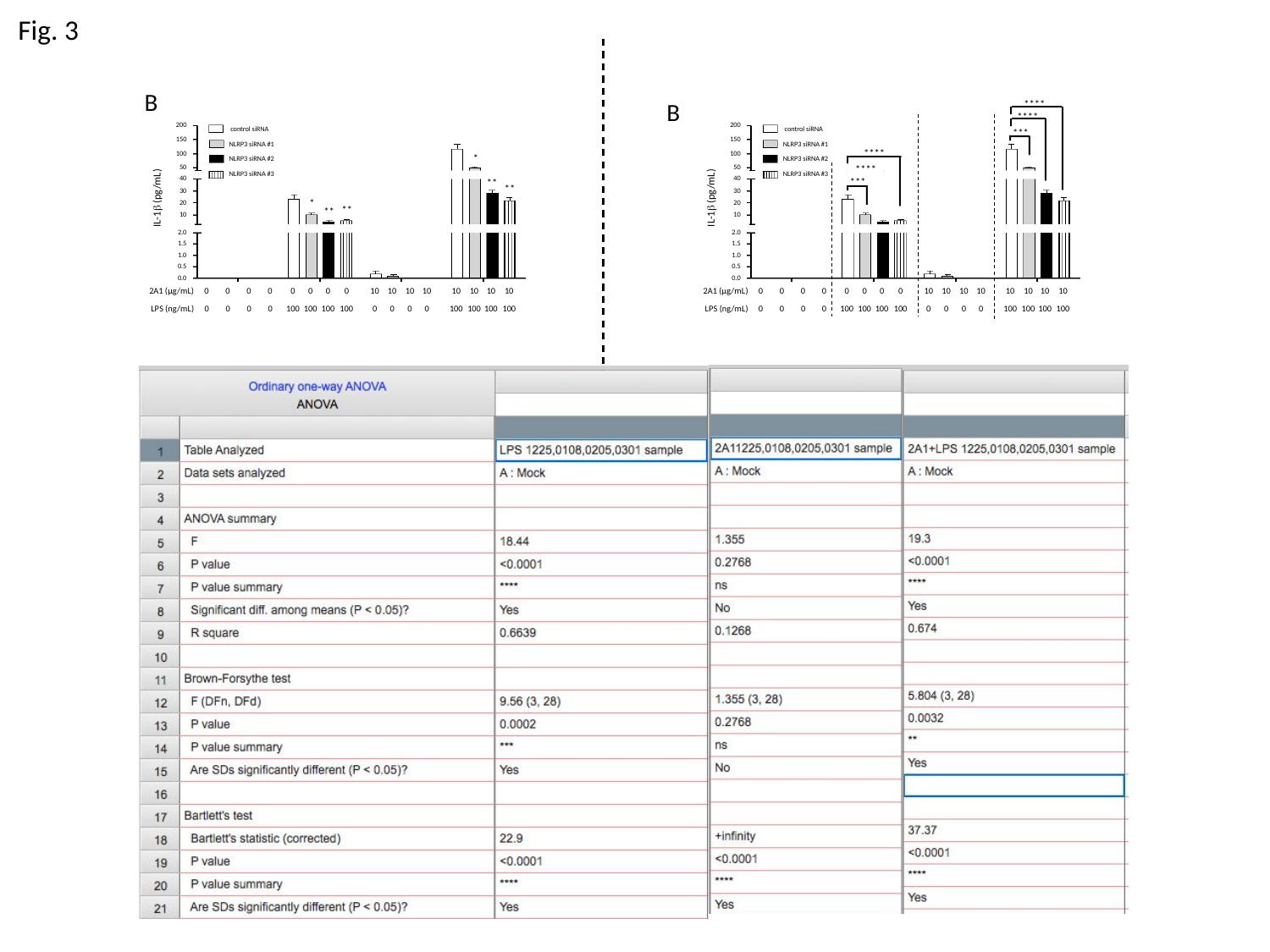

Fig. 3
B
B
****
****
200
control siRNA
NLRP3 siRNA #1
NLRP3 siRNA #2
NLRP3 siRNA #3
***
150
****
100
****
50
40
***
30
IL-1β (pg/mL)
20
10
2.0
1.5
1.0
0.5
0.0
2A1 (μg/mL)
0
0
0
0
0
0
0
0
10
10
10
10
10
10
10
10
LPS (ng/mL)
0
0
0
0
100
100
100
100
0
0
0
0
100
100
100
100
200
control siRNA
NLRP3 siRNA #1
NLRP3 siRNA #2
NLRP3 siRNA #3
150
100
*
50
40
**
**
30
IL-1β (pg/mL)
*
20
**
**
10
2.0
1.5
1.0
0.5
0.0
2A1 (μg/mL)
0
0
0
0
0
0
0
0
10
10
10
10
10
10
10
10
LPS (ng/mL)
0
0
0
0
100
100
100
100
0
0
0
0
100
100
100
100

## Slide 10
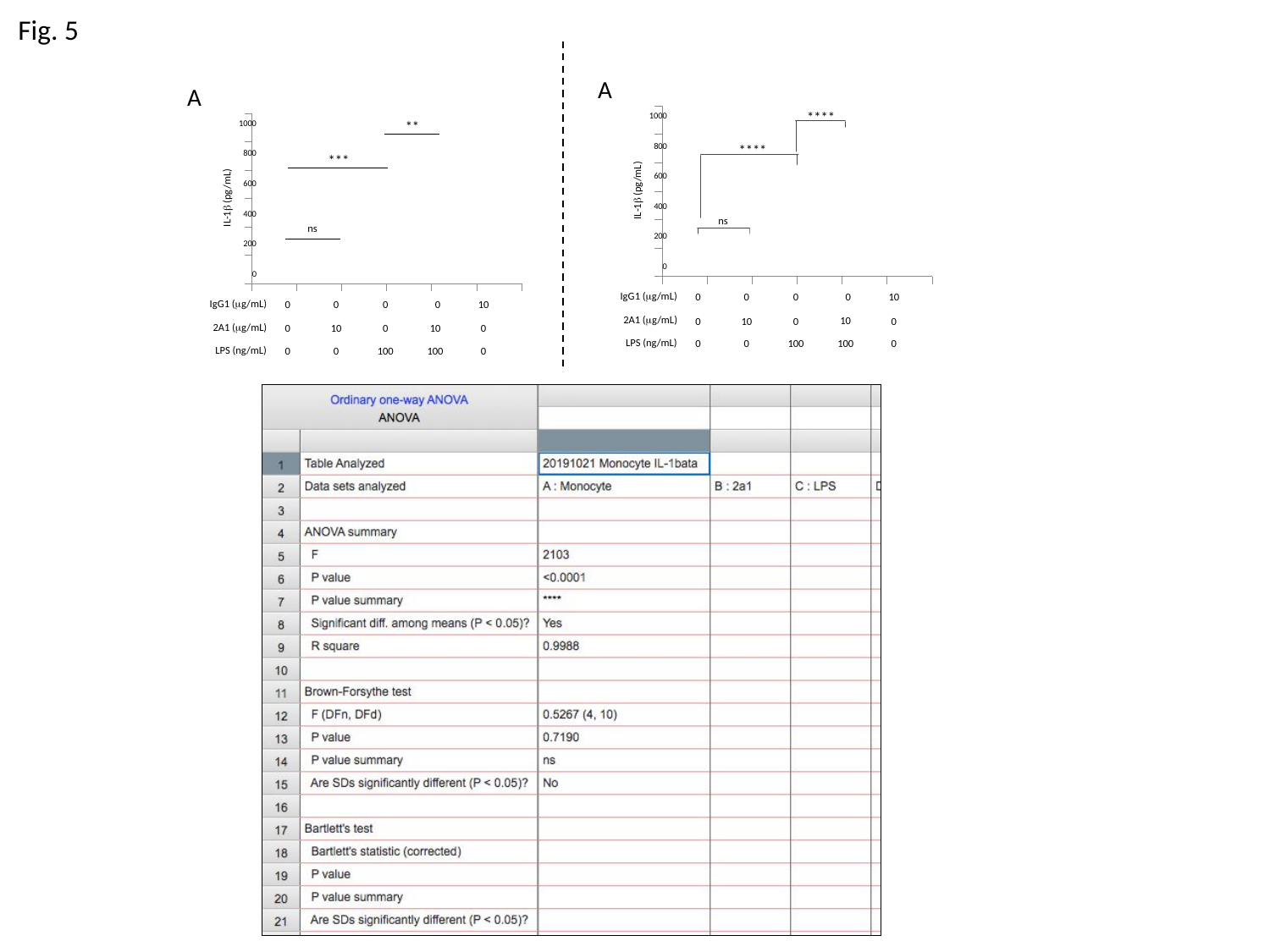

Fig. 5
A
### Chart
| Category | |
|---|---|
| Monocyte | 168.35839999999996 |
| 2a1 | 188.3584 |
| 2A1+LPS | 636.3584 |
| LPS | 843.3583999999998 |
| IgG | 81.35839999999999 |1000
800
600
IL-1β (pg/mL)
400
200
0
IgG1 (μg/mL)
0
0
0
0
10
2A1 (μg/mL)
10
0
10
0
0
LPS (ng/mL)
0
0
100
100
0
****
****
ns
A
### Chart
| Category | |
|---|---|
| Monocyte | 168.3584 |
| 2a1 | 188.3584 |
| 2A1+LPS | 636.3584 |
| LPS | 843.3583999999994 |
| IgG | 81.35839999999993 |1000
800
600
IL-1β (pg/mL)
400
200
0
IgG1 (μg/mL)
0
0
0
0
10
2A1 (μg/mL)
10
0
10
0
0
LPS (ng/mL)
0
0
100
100
0
**
***
ns

## Slide 11
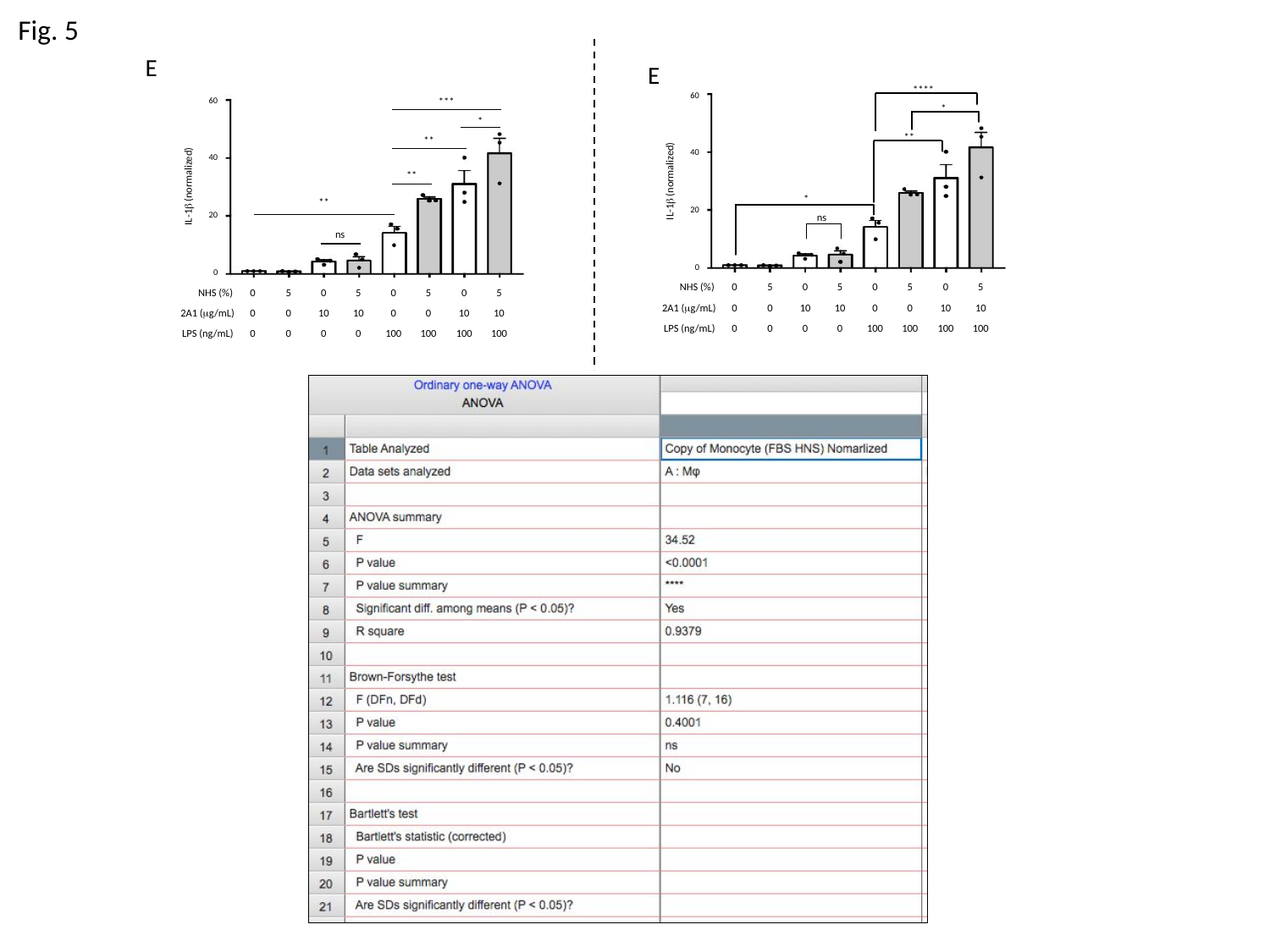

Fig. 5
E
E
****
60
*
**
40
IL-1β (normalized)
*
20
ns
0
NHS (%)
0
5
0
5
0
5
0
5
2A1 (μg/mL)
0
0
10
10
0
0
10
10
LPS (ng/mL)
0
0
0
0
100
100
100
100
***
60
*
**
40
IL-1β (normalized)
**
20
ns
0
NHS (%)
0
5
0
5
0
5
0
5
2A1 (μg/mL)
0
0
10
10
0
0
10
10
LPS (ng/mL)
0
0
0
0
100
100
100
100
**

## Slide 12
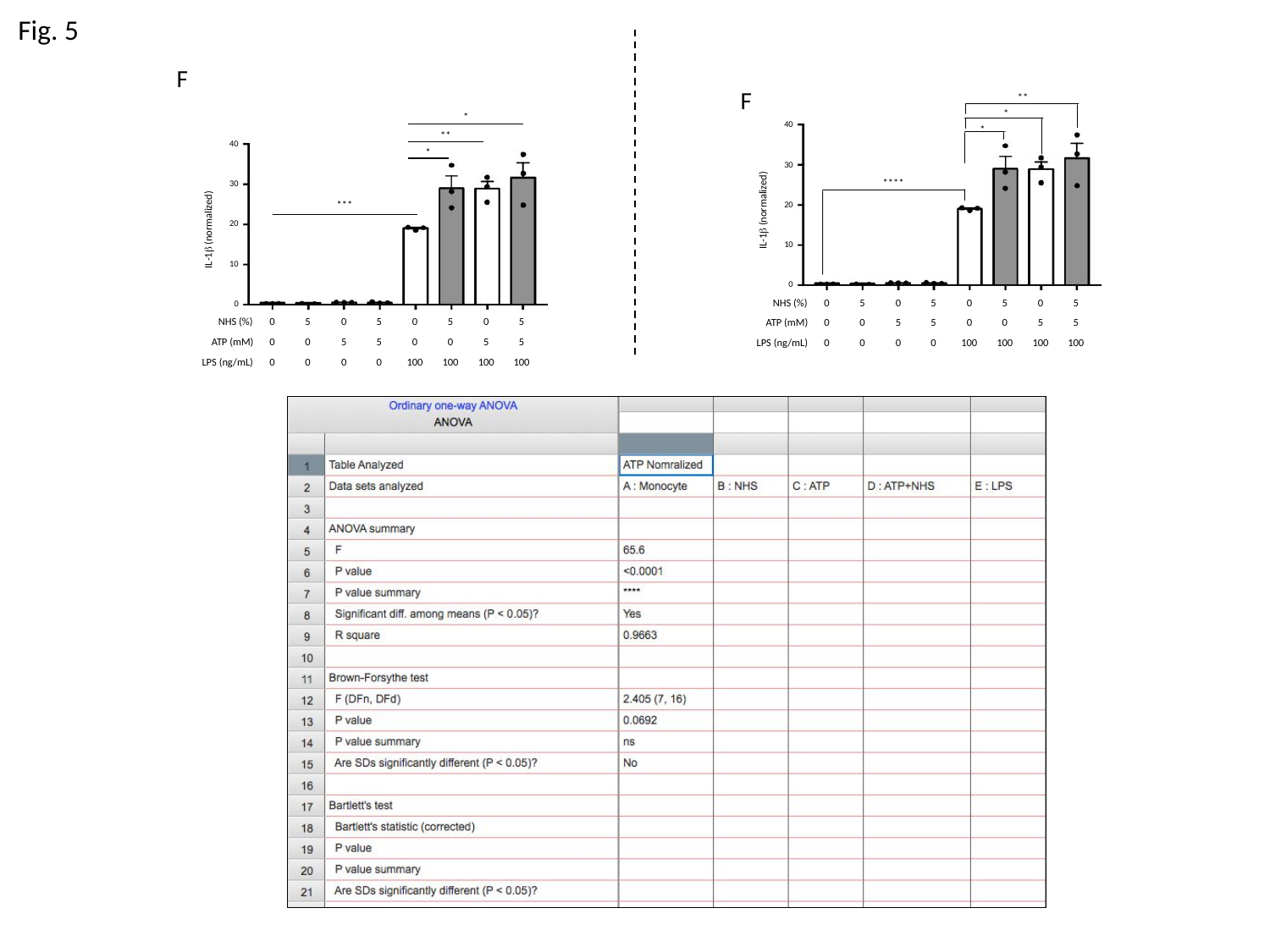

Fig. 5
F
F
**
*
40
*
30
****
IL-1β (normalized)
20
10
0
NHS (%)
0
5
0
5
0
5
0
5
ATP (mM)
0
0
5
5
0
0
5
5
LPS (ng/mL)
0
0
0
0
100
100
100
100
*
**
40
*
30
***
IL-1β (normalized)
20
10
0
NHS (%)
0
5
0
5
0
5
0
5
ATP (mM)
0
0
5
5
0
0
5
5
LPS (ng/mL)
0
0
0
0
100
100
100
100

## Slide 13
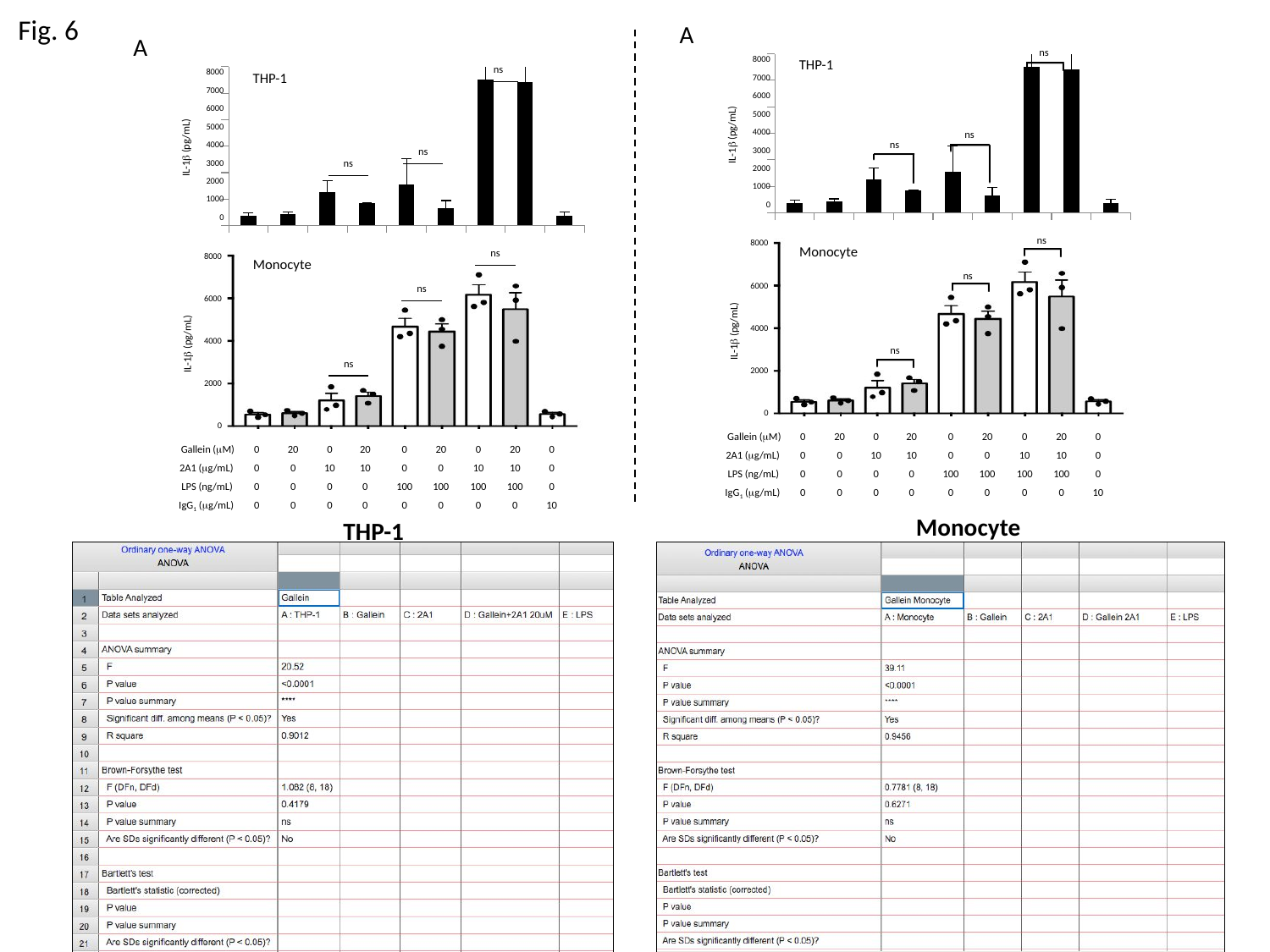

Fig. 6
A
ns
### Chart
| Category | |
|---|---|
| THP-1 | 350.162962962963 |
| Gallein | 414.0148148148149 |
| 2A1 | 1248.792592592593 |
| Gallein+2A1 20uM | 835.5703703703705 |
| LPS | 1545.348148148148 |
| Gallein+LPS | 653.0518518518519 |
| 2A1+LPS | 5521.903703703703 |
| Gallein+LPS+2A1 | 5427.274074074075 |
| IgG | 367.6444444444444 |8000
THP-1
7000
6000
5000
IL-1β (pg/mL)
4000
ns
ns
3000
2000
1000
0
ns
8000
Monocyte
ns
6000
IL-1β (pg/mL)
4000
ns
2000
0
Gallein (μM)
0
20
0
20
0
20
0
20
0
2A1 (μg/mL)
0
0
10
10
0
0
10
10
0
LPS (ng/mL)
0
0
0
0
100
100
100
100
0
IgG1 (μg/mL)
0
0
0
0
0
0
0
0
10
A
### Chart
| Category | |
|---|---|
| THP-1 | 350.162962962963 |
| Gallein | 414.0148148148149 |
| 2A1 | 1248.792592592593 |
| Gallein+2A1 20uM | 835.5703703703705 |
| LPS | 1545.348148148148 |
| Gallein+LPS | 653.0518518518519 |
| 2A1+LPS | 5521.903703703703 |
| Gallein+LPS+2A1 | 5427.274074074075 |
| IgG | 367.6444444444444 |ns
8000
7000
6000
5000
4000
ns
ns
3000
2000
1000
0
IL-1β (pg/mL)
THP-1
ns
8000
ns
6000
IL-1β (pg/mL)
4000
ns
2000
0
Gallein (μM)
0
20
0
20
0
20
0
20
0
2A1 (μg/mL)
0
0
10
10
0
0
10
10
0
LPS (ng/mL)
0
0
0
0
100
100
100
100
0
IgG1 (μg/mL)
0
0
0
0
0
0
0
0
10
Monocyte
 Monocyte
 THP-1

## Slide 14
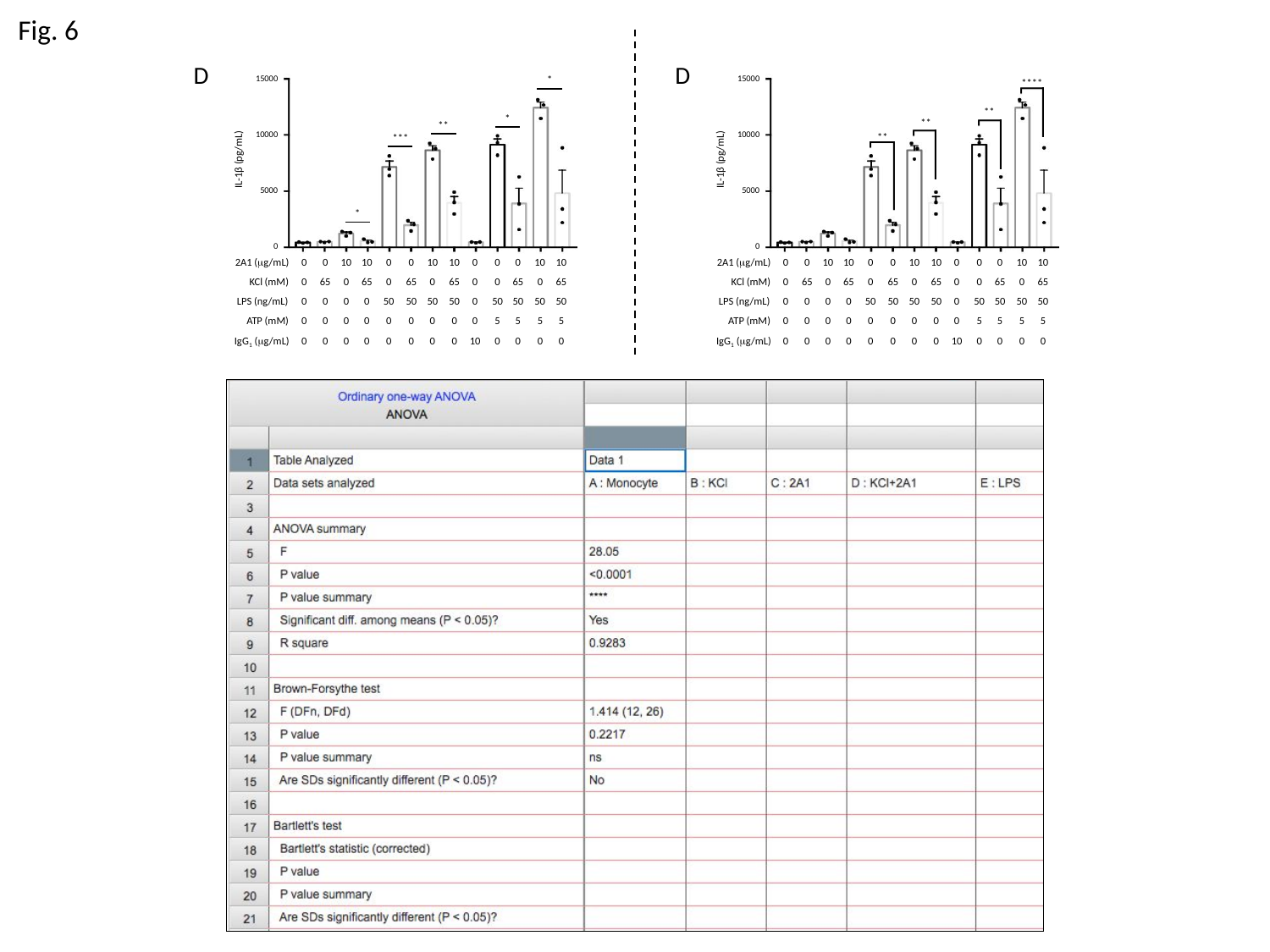

Fig. 6
D
D
15000
****
**
**
10000
**
IL-1β (pg/mL)
5000
0
2A1 (μg/mL)
0
0
10
10
0
0
10
10
0
0
0
10
10
KCl (mM)
0
65
0
65
0
65
0
65
0
0
65
0
65
LPS (ng/mL)
0
0
0
0
50
50
50
50
0
50
50
50
50
ATP (mM)
0
0
0
0
0
0
0
0
0
5
5
5
5
IgG1 (μg/mL)
0
0
0
0
0
0
0
0
10
0
0
0
0
15000
*
*
**
10000
***
IL-1β (pg/mL)
5000
*
0
2A1 (μg/mL)
0
0
10
10
0
0
10
10
0
0
0
10
10
KCl (mM)
0
65
0
65
0
65
0
65
0
0
65
0
65
LPS (ng/mL)
0
0
0
0
50
50
50
50
0
50
50
50
50
ATP (mM)
0
0
0
0
0
0
0
0
0
5
5
5
5
IgG1 (μg/mL)
0
0
0
0
0
0
0
0
10
0
0
0
0

## Slide 15
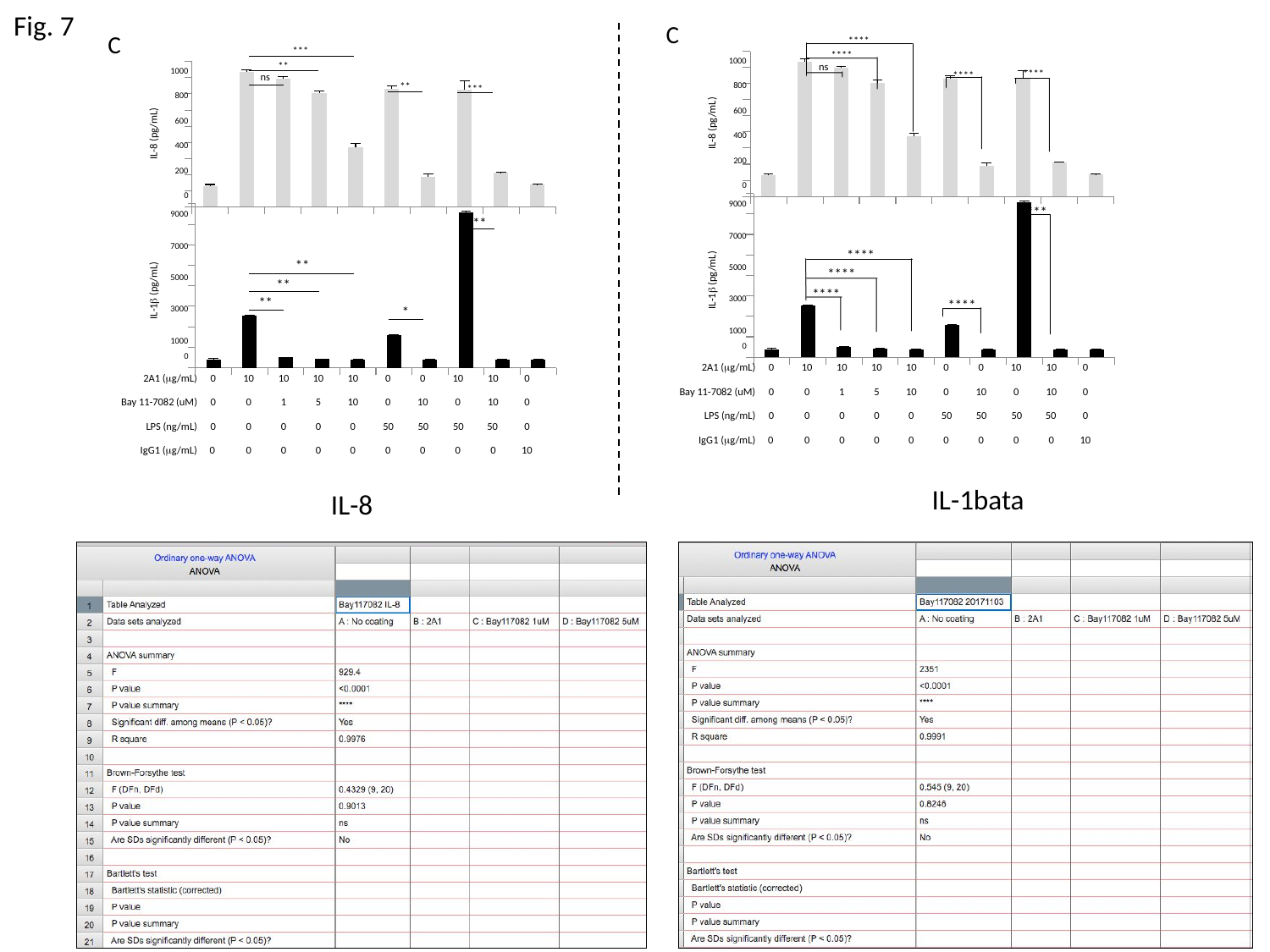

Fig. 7
C
C
****
***
### Chart
| Category | |
|---|---|
| No coating | 129.75 |
| 2A1 | 833.5 |
| Bay117082 1uM | 796.0 |
| Bay117082 5uM | 705.9999999999999 |
| Bay117082 10uM | 371.0 |
| LPS | 732.25 |
| LPS+Bay1170 | 187.25 |
| 2A1+LPS | 727.25 |
| 2A1+LPS+Bay117082 | 211.0 |
| IgG | 137.25 |**
1000
ns
**
***
800
600
IL-8 (pg/mL)
400
200
0
### Chart
| Category | |
|---|---|
| No coating | 390.4 |
| 2A1 | 2538.4 |
| Bay117082 1uM | 502.4 |
| Bay117082 5uM | 422.4 |
| Bay117082 10uM | 402.4 |
| LPS | 1598.4 |
| LPS+Bay1170 | 382.4 |
| 2A1+LPS | 7590.4 |
| 2A1+LPS+Bay117082 | 398.4 |
| IgG | 398.4 |9000
***
7000
**
5000
**
IL-1β (pg/mL)
**
*
3000
1000
0
0
10
10
10
10
0
0
10
10
0
2A1 (μg/mL)
0
0
1
5
10
0
Bay 11-7082 (uM)
0
10
0
10
0
0
0
0
0
50
50
50
50
0
LPS (ng/mL)
0
0
0
0
0
0
0
0
0
10
IgG1 (μg/mL)
### Chart
| Category | |
|---|---|
| No coating | 129.75 |
| 2A1 | 833.5 |
| Bay117082 1uM | 796.0 |
| Bay117082 5uM | 705.9999999999999 |
| Bay117082 10uM | 371.0 |
| LPS | 732.25 |
| LPS+Bay1170 | 187.25 |
| 2A1+LPS | 727.25 |
| 2A1+LPS+Bay117082 | 211.0 |
| IgG | 137.25 |****
1000
ns
****
****
800
600
IL-8 (pg/mL)
400
200
0
### Chart
| Category | |
|---|---|
| No coating | 390.4 |
| 2A1 | 2538.4 |
| Bay117082 1uM | 502.4 |
| Bay117082 5uM | 422.4 |
| Bay117082 10uM | 402.4 |
| LPS | 1598.4 |
| LPS+Bay1170 | 382.4 |
| 2A1+LPS | 7590.4 |
| 2A1+LPS+Bay117082 | 398.4 |
| IgG | 398.4 |9000
****
7000
****
5000
****
IL-1β (pg/mL)
****
3000
****
1000
0
0
10
10
10
10
0
0
10
10
0
2A1 (μg/mL)
0
0
1
5
10
0
Bay 11-7082 (uM)
0
10
0
10
0
0
0
0
0
50
50
50
50
0
LPS (ng/mL)
0
0
0
0
0
0
0
0
0
10
IgG1 (μg/mL)
IL-1bata
IL-8

## Slide 16
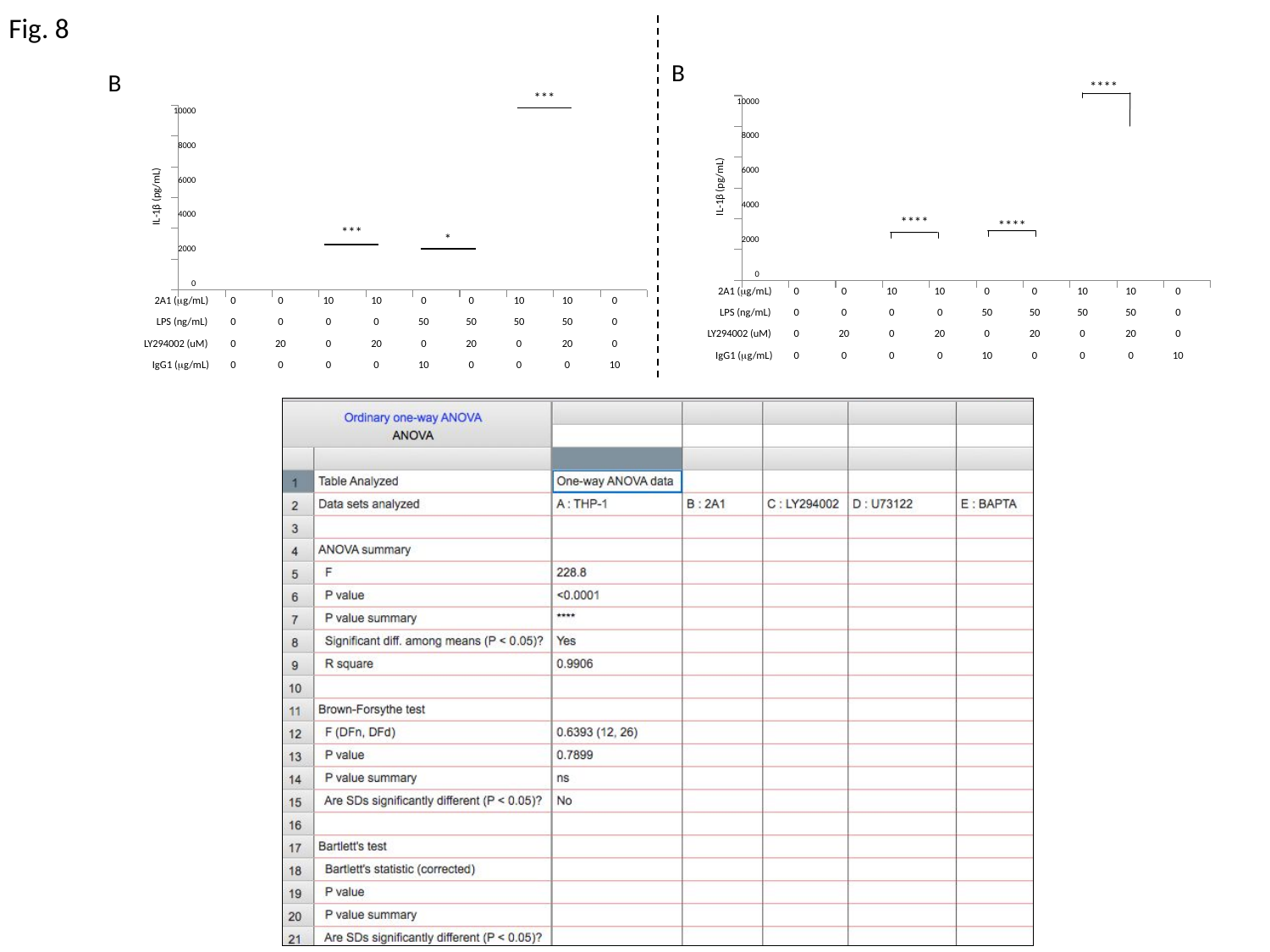

Fig. 8
B
****
### Chart
| Category | |
|---|---|
| THP-1 | 505.999999999999 |
| LY294002 | 549.3333333333333 |
| 2A1 | 1502.666666666667 |
| LY294002+2A1 | 592.6666666666666 |
| LPS | 1219.333333333333 |
| LY294002+LPS | 909.3333333333333 |
| 2A1+LPS | 9369.333333333325 |
| LY294002+2A1+LPS | 7526.0 |
| IgG | 589.3333333333333 |10000
8000
6000
IL-1β (pg/mL)
4000
****
****
2000
0
2A1 (μg/mL)
0
0
10
10
0
0
10
10
0
LPS (ng/mL)
0
0
0
0
50
50
50
50
0
LY294002 (uM)
0
20
0
20
0
20
0
20
0
IgG1 (μg/mL)
0
0
0
0
10
0
0
0
10
B
***
### Chart
| Category | |
|---|---|
| THP-1 | 505.999999999999 |
| LY294002 | 549.3333333333333 |
| 2A1 | 1502.666666666667 |
| LY294002+2A1 | 592.6666666666666 |
| LPS | 1219.333333333333 |
| LY294002+LPS | 909.3333333333333 |
| 2A1+LPS | 9369.333333333325 |
| LY294002+2A1+LPS | 7526.0 |
| IgG | 589.3333333333333 |10000
8000
6000
IL-1β (pg/mL)
4000
***
*
2000
0
2A1 (μg/mL)
0
0
10
10
0
0
10
10
0
LPS (ng/mL)
0
0
0
0
50
50
50
50
0
LY294002 (uM)
0
20
0
20
0
20
0
20
0
IgG1 (μg/mL)
0
0
0
0
10
0
0
0
10

## Slide 17
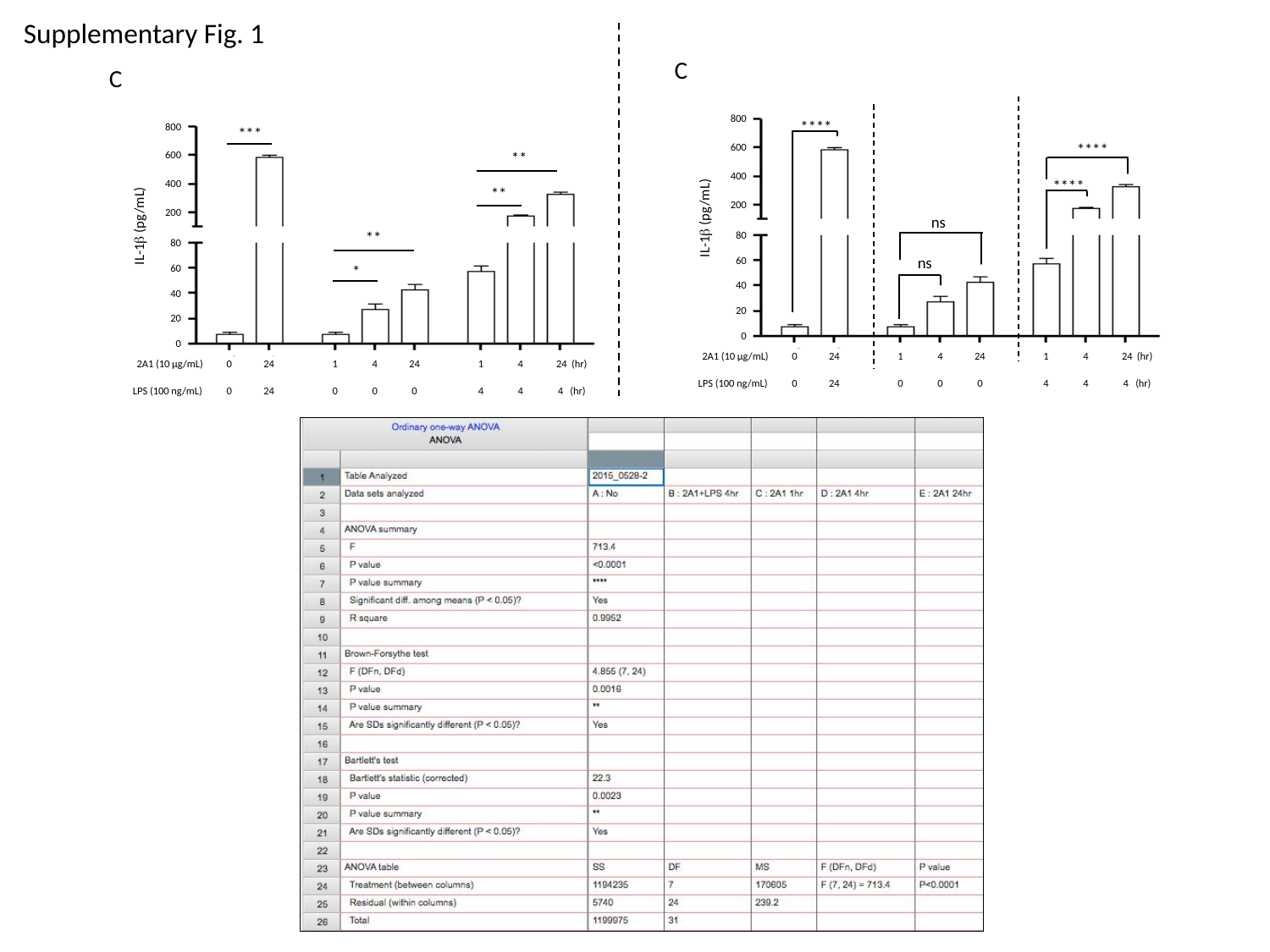

Supplementary Fig. 1
C
800
****
****
600
400
****
200
IL-1β (pg/mL)
ns
80
ns
60
40
20
0
2A1 (10 μg/mL)
0
24
1
4
24
1
4
24 (hr)
LPS (100 ng/mL)
0
24
0
0
0
4
4
4 (hr)
C
800
**
600
400
**
200
IL-1β (pg/mL)
**
80
*
60
40
20
0
2A1 (10 μg/mL)
0
24
1
4
24
1
4
24 (hr)
LPS (100 ng/mL)
0
24
0
0
0
4
4
4 (hr)
***

## Slide 18
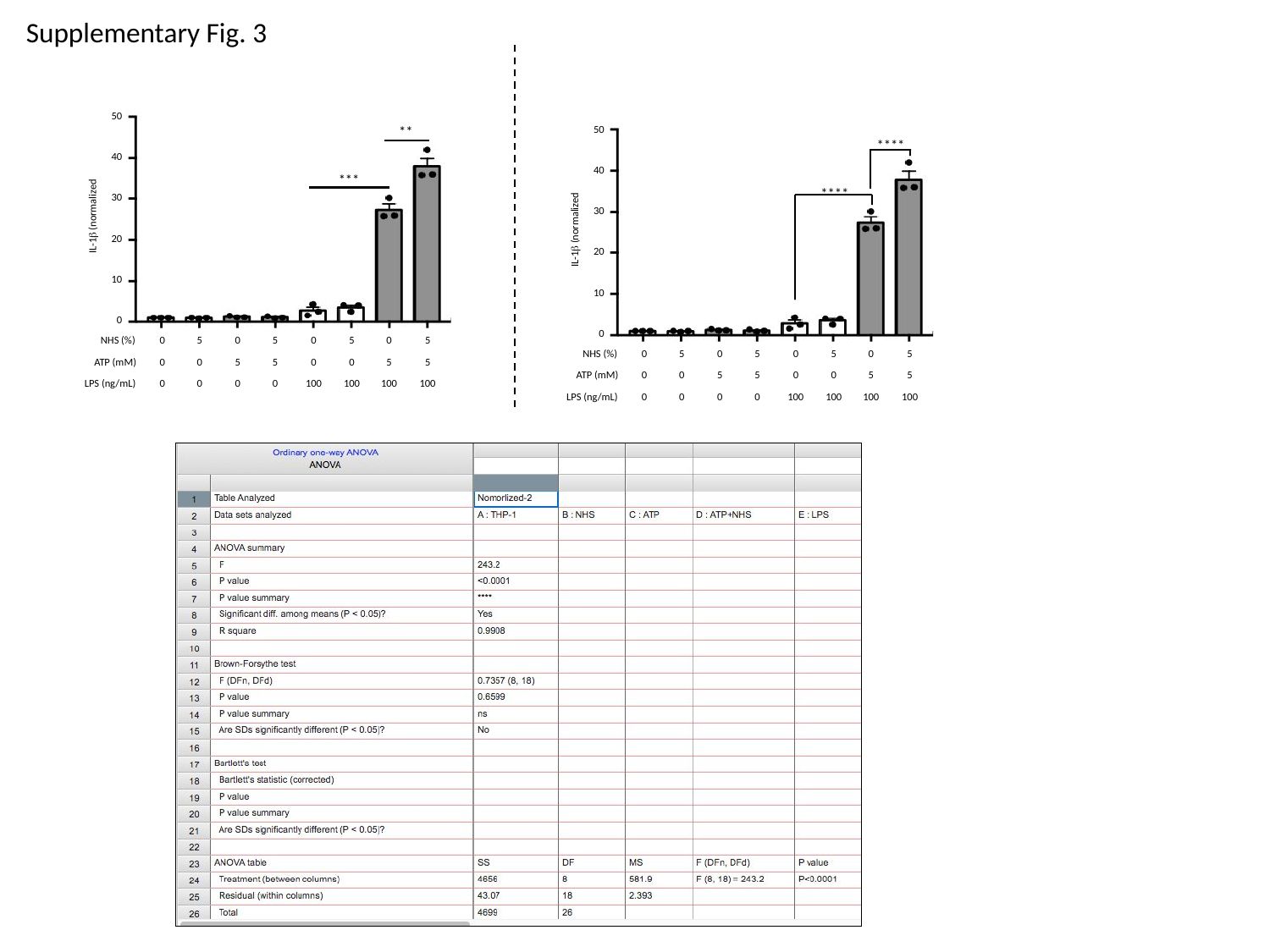

Supplementary Fig. 3
50
**
40
***
30
IL-1β (normalized
20
10
0
NHS (%)
0
5
0
5
0
5
0
5
ATP (mM)
0
0
5
5
0
0
5
5
LPS (ng/mL)
0
0
0
0
100
100
100
100
50
****
40
****
30
IL-1β (normalized
20
10
0
NHS (%)
0
5
0
5
0
5
0
5
ATP (mM)
0
0
5
5
0
0
5
5
LPS (ng/mL)
0
0
0
0
100
100
100
100

## Slide 19
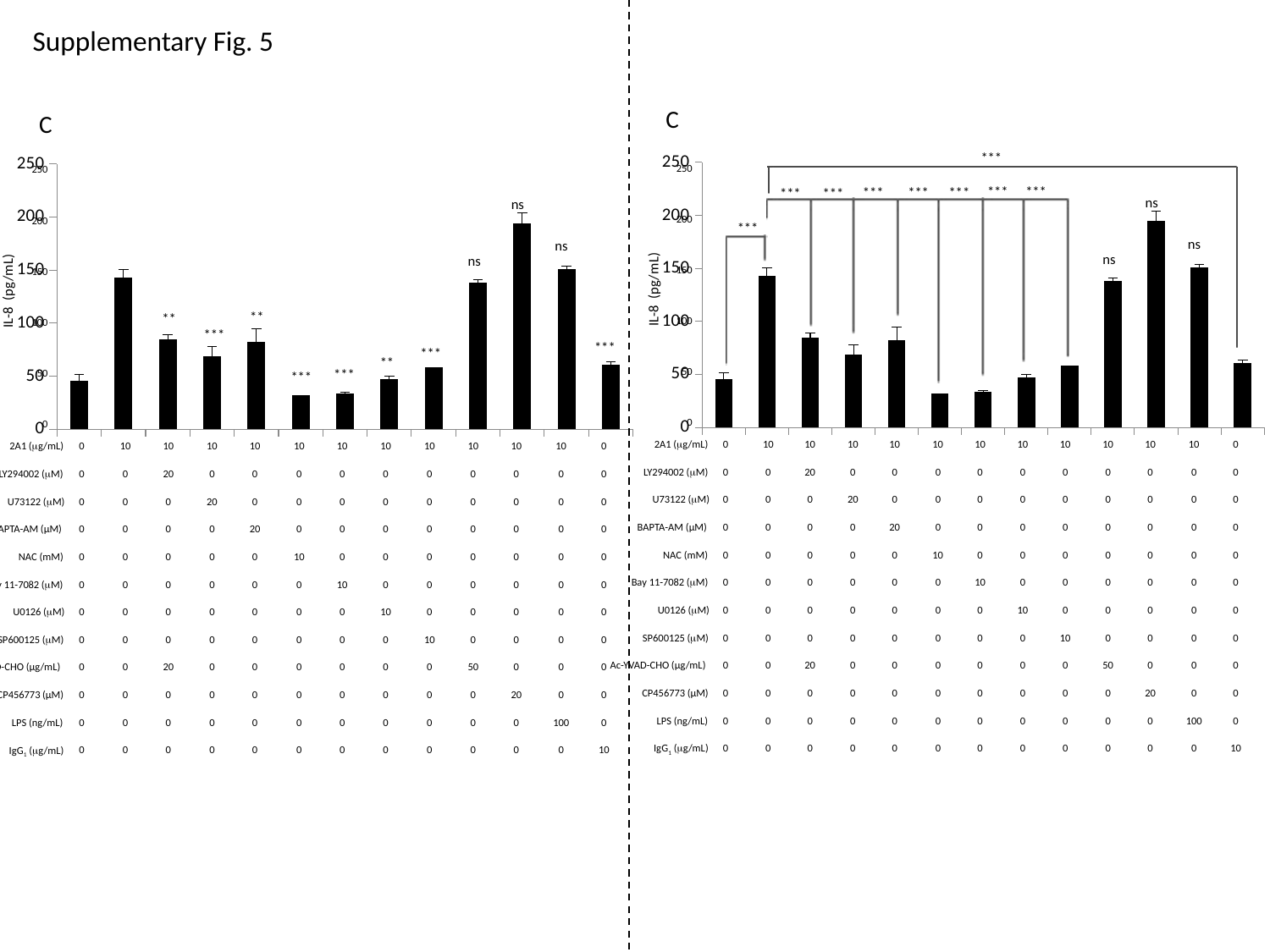

Supplementary Fig. 5
C
***
### Chart
| Category | |
|---|---|
| THP-1 | 45.48888888888889 |
| 2A1 | 143.26666666666665 |
| LY294002 | 85.04444444444445 |
| U73122 | 68.82222222222222 |
| BAPTA | 82.15555555555555 |
| NAC | 31.933333333333326 |
| Bay117082 | 33.93333333333333 |
| U0126 | 47.26666666666666 |
| sp600125 | 57.933333333333316 |
| Ac-YVAD-CHO | 138.15555555555557 |
| cp345773 | 194.37777777777782 |
| LPS | 150.60000000000002 |
| IgG | 60.822222222222216 |250
200
150
IL-8 (pg/mL)
100
50
0
2A1 (μg/mL)
0
10
10
10
10
10
10
10
10
10
10
10
0
LY294002 (μM)
0
0
20
0
0
0
0
0
0
0
0
0
0
U73122 (μM)
0
0
0
20
0
0
0
0
0
0
0
0
0
BAPTA-AM (μM)
0
0
0
0
20
0
0
0
0
0
0
0
0
NAC (mM)
0
0
0
0
0
10
0
0
0
0
0
0
0
Bay 11-7082 (μM)
0
0
0
0
0
0
10
0
0
0
0
0
0
U0126 (μM)
0
0
0
0
0
0
0
10
0
0
0
0
0
SP600125 (μM)
0
0
0
0
0
0
0
0
10
0
0
0
0
Ac-YVAD-CHO (μg/mL)
0
0
20
0
0
0
0
0
0
50
0
0
0
CP456773 (μM)
0
0
0
0
0
0
0
0
0
0
20
0
0
LPS (ng/mL)
0
0
0
0
0
0
0
0
0
0
0
100
0
IgG1 (μg/mL)
0
0
0
0
0
0
0
0
0
0
0
0
10
***
***
***
***
***
***
***
ns
***
ns
ns
C
### Chart
| Category | |
|---|---|
| THP-1 | 45.48888888888889 |
| 2A1 | 143.2666666666667 |
| LY294002 | 85.04444444444447 |
| U73122 | 68.82222222222221 |
| BAPTA | 82.15555555555554 |
| NAC | 31.93333333333332 |
| Bay117082 | 33.93333333333333 |
| U0126 | 47.26666666666664 |
| sp600125 | 57.93333333333332 |
| Ac-YVAD-CHO | 138.1555555555556 |
| cp345773 | 194.3777777777778 |
| LPS | 150.6 |
| IgG | 60.82222222222222 |250
200
150
IL-8 (pg/mL)
100
50
0
2A1 (μg/mL)
0
10
10
10
10
10
10
10
10
10
10
10
0
LY294002 (μM)
0
0
20
0
0
0
0
0
0
0
0
0
0
U73122 (μM)
0
0
0
20
0
0
0
0
0
0
0
0
0
BAPTA-AM (μM)
0
0
0
0
20
0
0
0
0
0
0
0
0
NAC (mM)
0
0
0
0
0
10
0
0
0
0
0
0
0
Bay 11-7082 (μM)
0
0
0
0
0
0
10
0
0
0
0
0
0
U0126 (μM)
0
0
0
0
0
0
0
10
0
0
0
0
0
SP600125 (μM)
0
0
0
0
0
0
0
0
10
0
0
0
0
Ac-YVAD-CHO (μg/mL)
0
0
20
0
0
0
0
0
0
50
0
0
0
CP456773 (μM)
0
0
0
0
0
0
0
0
0
0
20
0
0
LPS (ng/mL)
0
0
0
0
0
0
0
0
0
0
0
100
0
IgG1 (μg/mL)
0
0
0
0
0
0
0
0
0
0
0
0
10
ns
ns
ns
**
**
***
***
***
**
***
***

## Slide 20
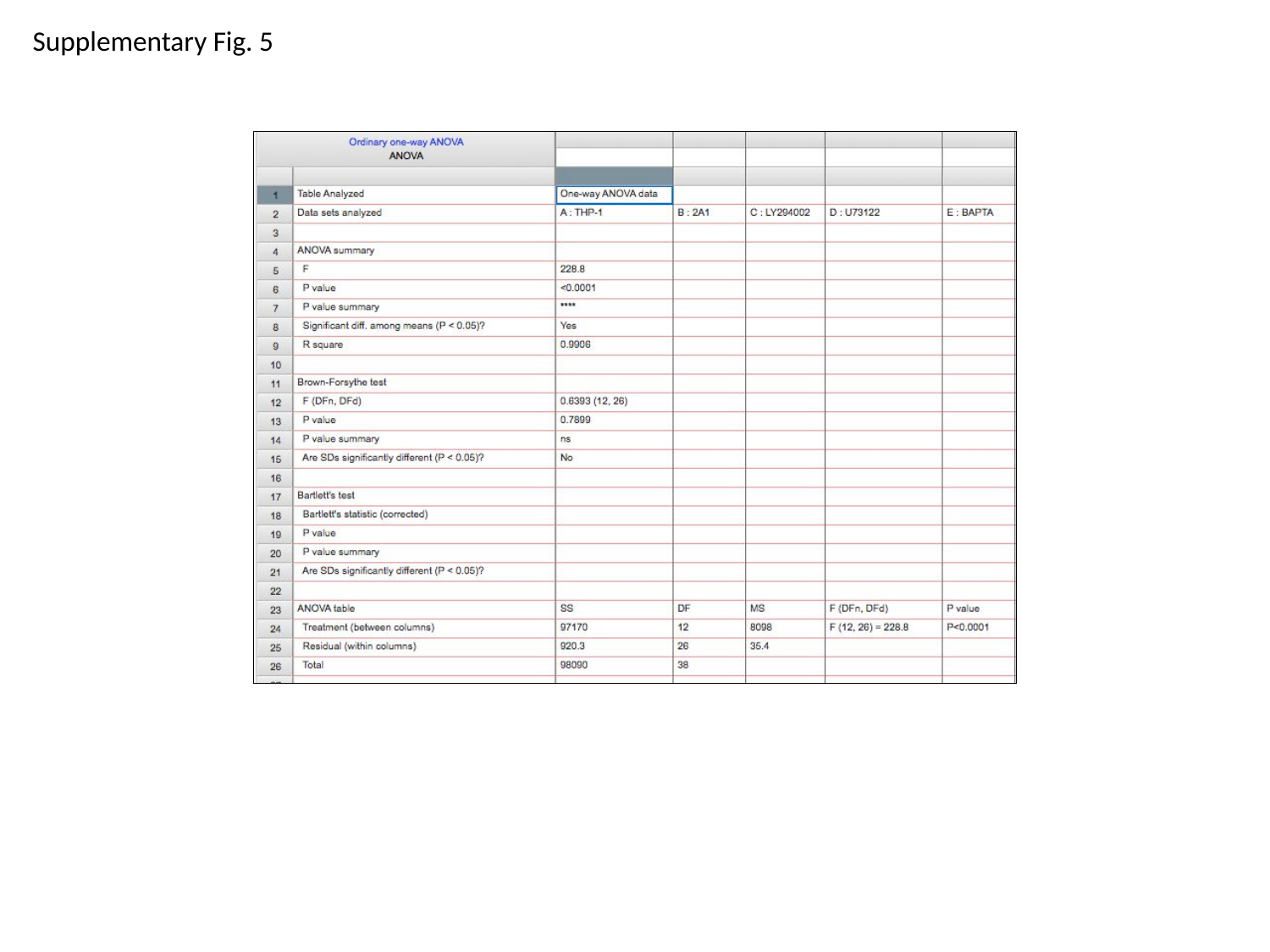

Supplementary Fig. 5
