# Supplementary figures and images for: Stimulation of Vibratory Urticaria-Associated Adhesion-GPCR, EMR2/ADGRE2, Triggers the NLRP3 Inflammasome Activation Signal in Human Monocytes
Source: Front Immunol. 2021 Jan 8;11:602016. doi: 10.3389/fimmu.2020.602016 (PMC7820815; doi:10.3389/fimmu.2020.602016)

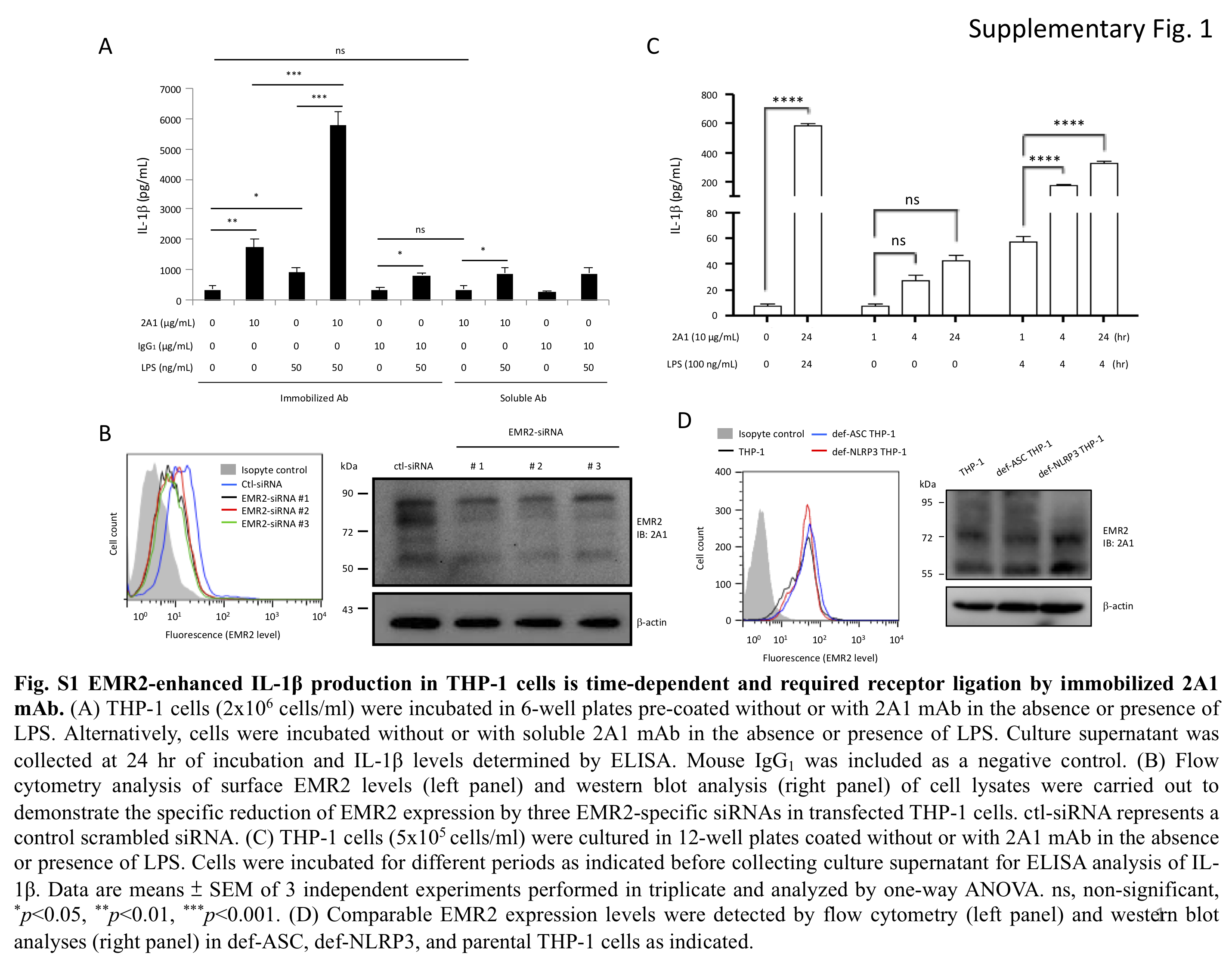

Supplement: Supplementary file 3 [file Image_1.tiff]

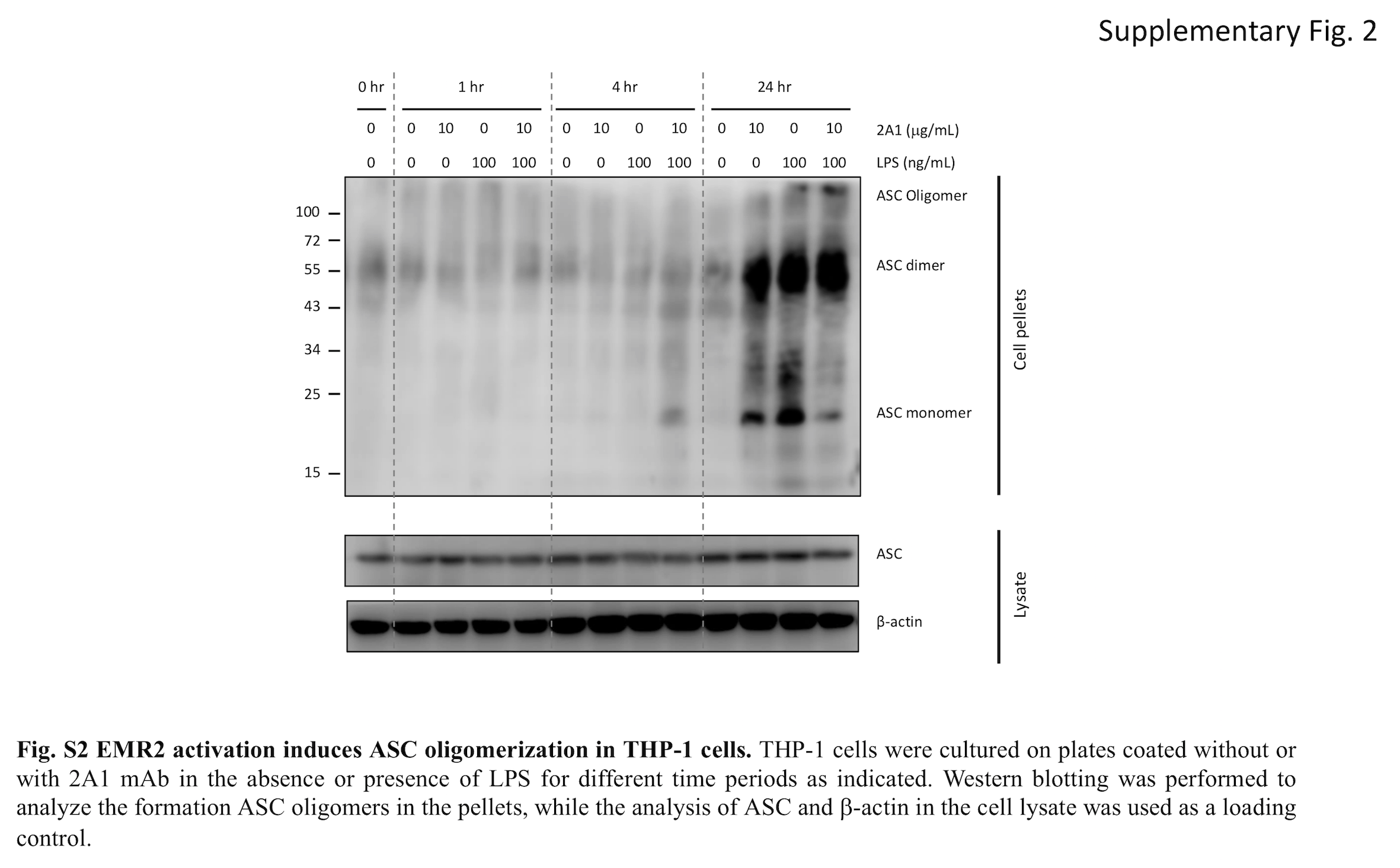

Supplement: Supplementary file 4 [file Image_2.tiff]

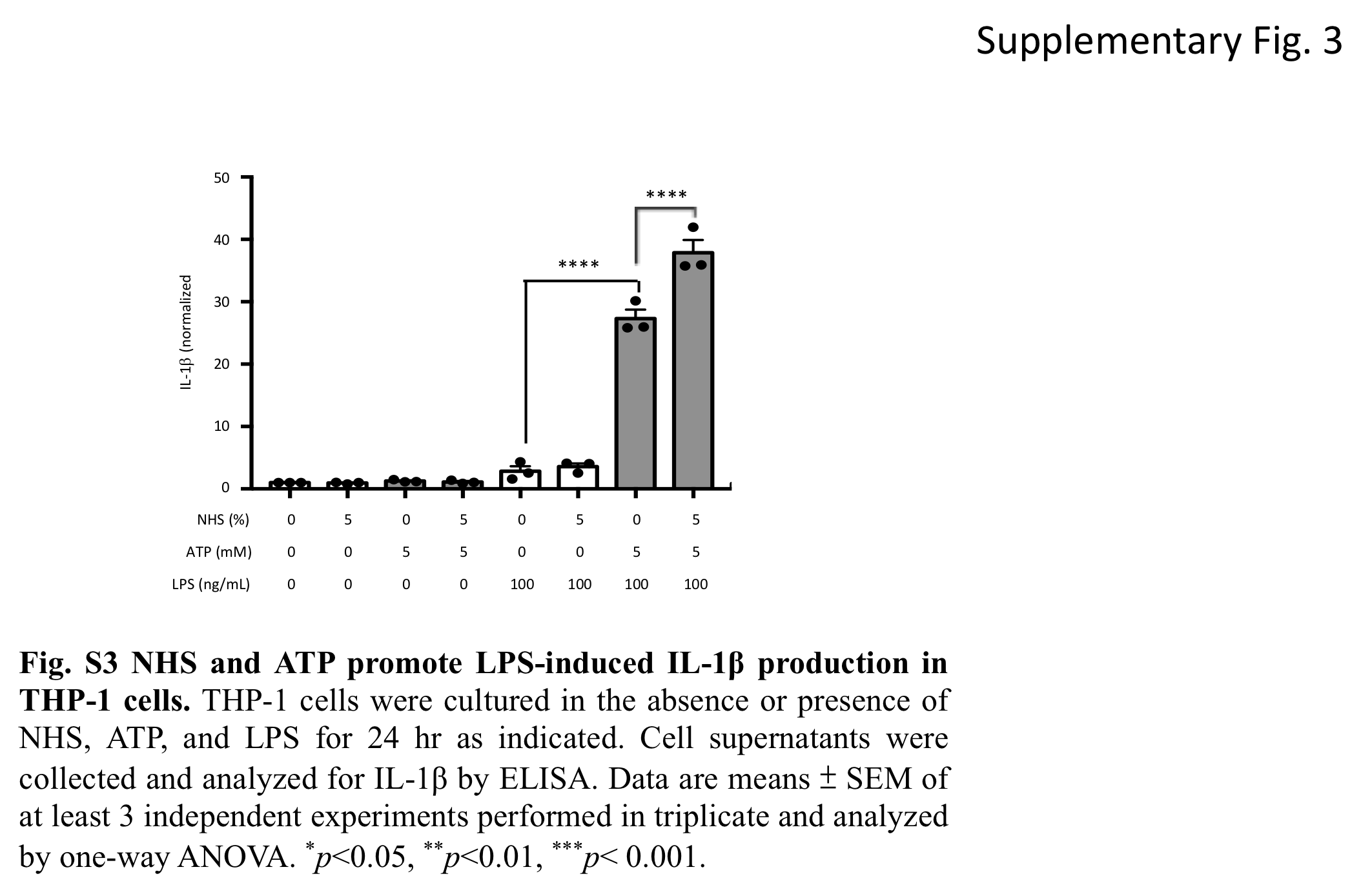

Supplement: Supplementary file 5 [file Image_3.tiff]

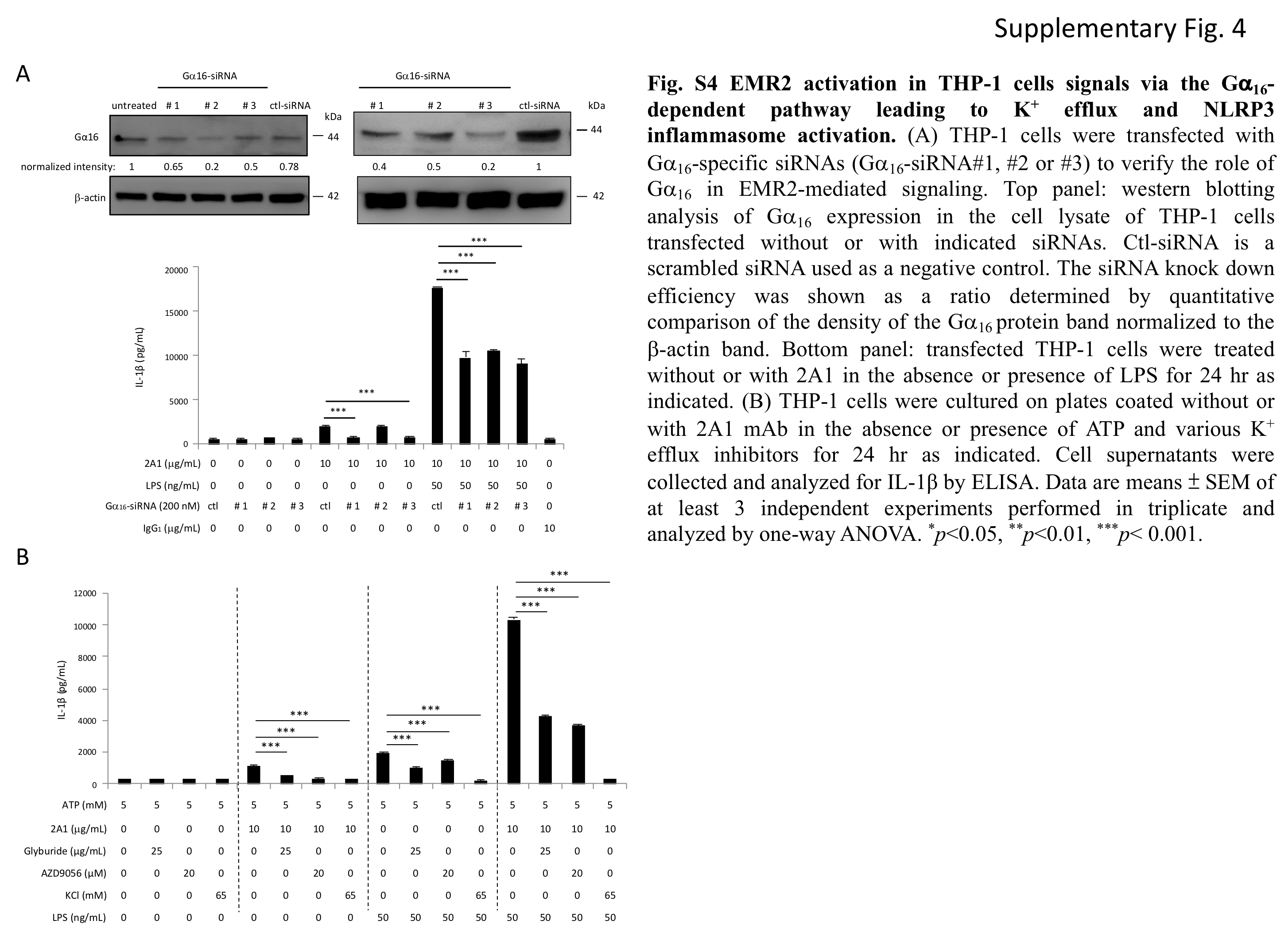

Supplement: Supplementary file 6 [file Image_4.tiff]

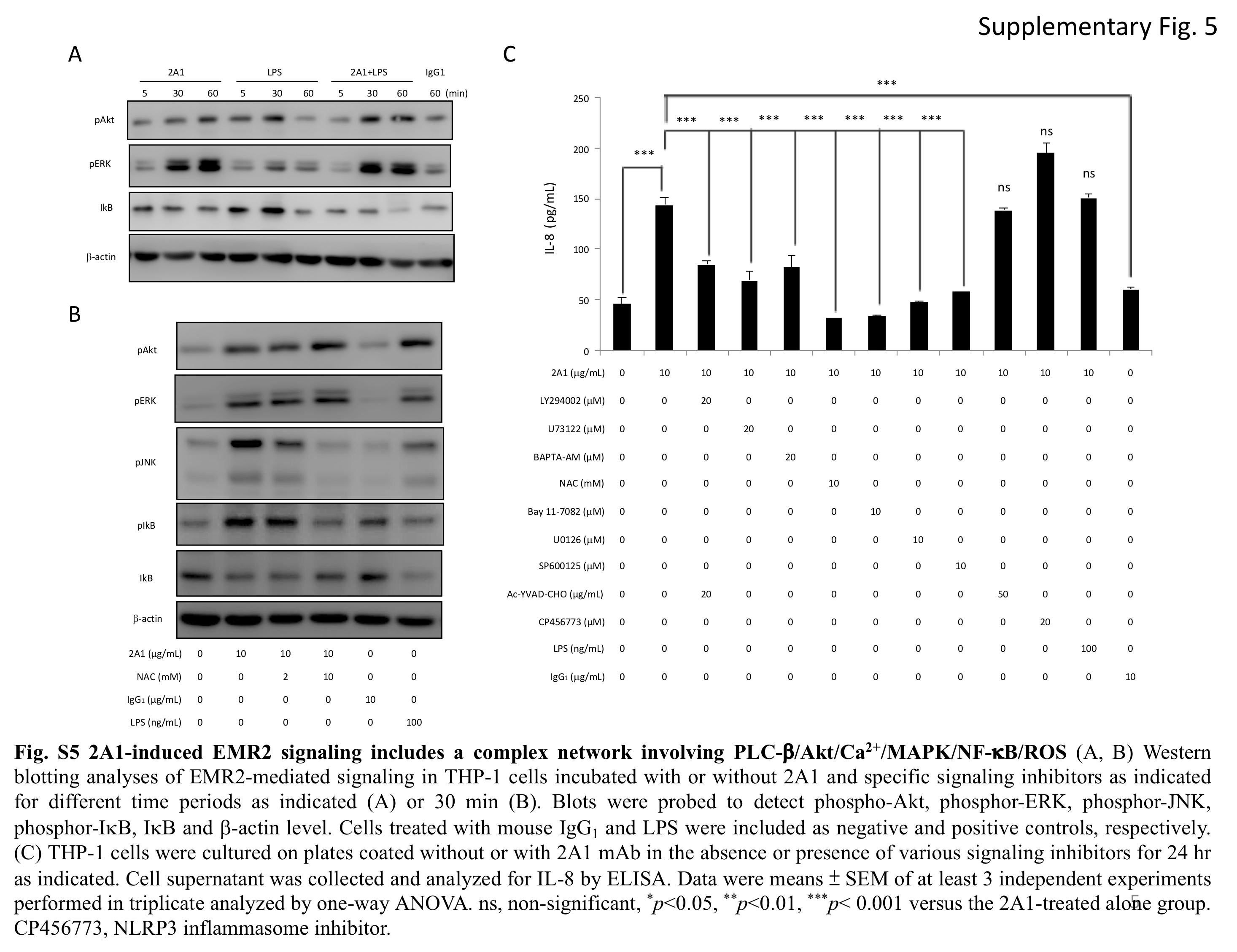

Supplement: Supplementary file 7 [file Image_5.tiff]
